# Neurocranium versus Face: A Morphometric Approach with Classical Anthropometric Variables for Characterizing Patterns of Cranial Integration in Extant Hominoids and Extinct Hominins

**DOI:** 10.1371/journal.pone.0131055

**Published:** 2015-07-15

**Authors:** Juan Antonio Pérez-Claros, Juan Manuel Jiménez-Arenas, Paul Palmqvist

**Affiliations:** 1 Departamento de Ecología y Geología, Facultad de Ciencias, Universidad de Málaga, Málaga, Spain; 2 Departamento de Prehistoria y Arqueología, Facultad de Filosofía y Letras, Universidad de Granada, Granada, Spain; University of Florence, ITALY

## Abstract

The relative importance of the two main cranial complexes, the neurocranium and the splanchnocranium, has been examined in the five species of extant hominoids and in a huge sample of extinct hominins using six standard craniometric variables that measure the length, width and height of each cranial module. Factor analysis and two-block partial least squares were used for establishing the major patterns of developmental and evolutionary integration between both cranial modules. The results obtained show that all extant hominoids (including the anatomically modern humans) share a conserved pattern of developmental integration, a result that agrees with previous studies. The pattern of evolutionary integration between both cranial modules in australopiths runs in parallel to developmental integration. In contrast, the pattern of evolutionary and developmental integration of the species of the genus *Homo* is the opposite, which is probably the consequence of distinctive selective regimes for both hominin groups.

## Introduction

The notion that those characters interacting in development and/or function tend to evolve concertedly is behind the concepts of modularity and morphological integration [1–4]. The two most prominent modules of the mammalian cranium are the neurocranium (i.e., the cerebral capsule or neurobasicranial complex) and the splanchnocranium (i.e., the face) [5–7]. These modules are inferred from both developmental processes and functional reasons (for a brief review, see [8]).

The evolutionary relationships between both cranial modules have attracted the attention of researchers since a long time (e.g., [9]), playing an increasing role in current studies on hominin evolution (e.g., [8]). Corruccini [10] was pioneer in assessing the morphometric relationships in extinct hominins between the face and the neurocranium from a multivariate point of view, indicating that a progressive reduction of the face run in parallel to an increase of the neurocranium. Lieberman [11] and Lieberman et al. [12] analyzed the differences in cranial morphology between the anatomically modern humans and other species of *Homo*. They found that the main differences are the presence in *H*. *sapiens* of a short sphenoid, a more rounded braincase and a reduced facial projection [12]. Similarly, Guy et al. [13] analyzed a taxonomically diverse sample of hominins in a search for the morphological affinities of *Sahelanthropus tchadensis*, with the obtaining of similar results. Specifically, they found that an important fraction of the variance (78%) spread along a multivariate vector that reflected the anteroposterior shortening of the rostrum, which was associated to an enlargement of the cranial vault. Finally, González-José et al. [14] found that, compared to other hominins, the clade that integrates the genus *Homo* is characterized by the presence of a more retracted face and an increase in neurocranial globularity. Therefore, there is consensus in accepting that the relative dimensions of the splanchnocranium and the neurocranium have changed noticeably during the evolution of hominins, which results from changes in the skull developmental program between the ancestors and their descendants.

Concerning the relationships between the cranial modules in hominoids, Chaline [15] proposed the existence of three discrete skull plans (namely, “great ape”, “australopithecine” and “*Homo*”). These skull plans would follow a morphological gradient characterized by a correlated increase in brain capacity and craniofacial contraction, which would be achieved by distinctive ontogenetic itineraries. However, Mitteroecker and Bookstein [8] pointed out that although there are substantial differences in cranial morphology among the extant species of hominoids, all them share the same major developmental processes and, consequently, show similar-but not identical- patterns of developmental integration. In fact, the analyses of Mitteroecker and Bookstein [8] showed that the clouds of points for the adult specimens of *Gorilla*, *Pan* and *Homo* have very similar orientations in the axes that account for the morphological covariation of the neurocranium and the viscerocranium. Similarly, Singh et al. [16] pointed out that both humans and apes show an overall similar pattern of integration between the face, the basicranium and the cranial vault.

The relationships between the development and evolution of the cranial modules can be also approached by their reflection in the patterns of intra- and interspecific covariation, respectively. In this regard, the position and orientation of the clouds of points in the size and shape space presented by Guy et al. [13] showed that the ontogenetic trajectories of *Gorilla*, *Pan* and *Homo* run more or less in parallel, although they were laterally transposed. This suggested that the evolutionary changes that took place within the hominoid clade were not the mere consequence of the truncation or extension of developmental trajectories in the stem lineages.

However, any study of the evolutionary patterns of covariation between the neurocranium and the splachnocranium that does not incorporate in the analyses representatives of extinct hominin species would be incomplete. In spite of the low preservational completeness of the hominin fossil record, our knowledge on the extinct hominins has increased spectacularly during the last decades due to the discovery of new taxa and the re-evaluation of the evidence already available [17–30]. This has resulted in a noticeable increase in the range of morphological, spatial and temporal variability of hominins. However, the relatively poor preservation of many fossil crania precludes applying to these taxa the standard, landmark-based techniques of geometric morphometrics, which would allow describing accurately the patterns of covariation between the neurocranium and the splachnocranium. This in turn prevents to perform a comparative study of cranial modularity and integration in the extant and extinct hominoids. In any case, it is possible to approach this issue from a different view. Much of the diversity in primate cranial morphology is closely related to the relative importance of their cranial modules [6] and consequently, any estimator of this might be considered as a valid starting point. One possible way for evaluating the relative importance of the cranial modules is to estimate their relative sizes, which can be easily achieved with the use of standard, “low-tech” metric variables and the methods of traditional morphometrics (e.g., principal components analysis and canonical discriminant functions). Given that this approach allows incorporating a relatively high number of fossils into the analyses, some authors [31] have preferred to choose among a limited number of osteological measurements instead of using other more efficient morphometric tools. Such approach can be reasonably appropriate when the study focus on the search for general patterns of craniofacial integration, although it could be inadequate for more detailed analyses. In addition, the modular nature of the cranium allows condensing many correlated (i.e., integrated) traits in a rather limited set of osteological variables instead of treating them as independent characters (e.g., [14]).

This article focuses on the search for intra- and interspecific patterns of covariation in the relative dimensions of the two main cranial modules in different subsets within the hominoid clade. We do not intend to identify these modules, as we assume their existence on the basis of previous studies (see references above). In order to increase the diversity of fossils in our analyses, the size of both cranial complexes were approached using only osteological variables that estimate the lengths of the cranium along the three directions of the space. Obviously, the inferences that can be obtained from this dataset relate only to the coarser aspects of cranial morphology. However, although this work schedule is relatively simple, it will enable us to analyze cranial morphology in the great apes, the modern humans and the two main groups of fossil hominins (i.e., australopiths and extinct members of *Homo*).

With this in mind, we are particularly interested in the following two questions: (1) does the modular nature of the cranium reflect the patterns of covariation among the length, width and height of each cranial module? And (2) if this were the case, are there different allometric rules for the relative size of the two main cranial modules? Our results indicate that although the relative size of each module is characteristic of each species, there is a common pattern of ontogenetic integration shared by all hominoids that can be detected, to a certain extent, using different methods. However, while the patterns of ontogenetic and evolutionary integration run in parallel for some groups (e.g., the African apes and the australopiths), in the case of the extinct members of the genus *Homo* these patterns run in an opposite direction, which probably reflects the existence of distinctive selective regimes, as discussed below.

## Materials and Methods

Our sample consists of adult specimens of the five extant hominoid species, *Pongo pygmaeus*, *Gorilla gorilla*, *Pan troglodytes* (three subspecies: *P*. *troglodytes troglodytes*, *P*. *troglodytes verus* and *P*. *troglodytes schweinfurthii*), *P*. *paniscus*, and *Homo sapiens* (Table 1). In the case of *H*. *sapiens*, several toothless individuals and two microcephalics were also included.

**Table 1:** Specimens used in this study for the species of living hominoids and extinct hominins sampled.

Group	n	Collection / References
AMH (recent)	141	PALUG; [32–35]
AMH (Pleistocene)	20	[22, 36–38]
AMH (recent toothless)	13	AIMUZ
AMH (recent microcephalic)	2	AIMUZ; PALUG; [39– 40]
*Pan paniscus*	20	RMCA
*Pan troglodytes*	54	AIMUZ; RMCA
*Gorilla gorilla*	29	AIMUZ
Pongo pygmaeus	14	AIMUZ
Fossil hominins	28	see Table 2

AIMUZ = Anthropological Institute and Museum, University of Zurich. RMCA = Royal Museum of Central Africa, Tervuren, Belgium. PALUG = Physical Anthropology Laboratory, University of Granada, Spain.

Provenance of the cranial specimens measured:

Anatomically modern *Homo sapiens* (AMH): AMH population from Tohoku (Japan), measurements taken from [32]. Specimens: THK 1, THK 2, THK 62, THK 256, THK 266, THK 281, THK 283, THK 287, THK 320, THK 349, THK 364, THK 376, THK 434, THK 1058, THK 1299, THK 1742, THK 2544, THK 2564. AMH population from La Torrecilla (Spain), a mediaeval cemetery. PALUG Collection. Specimens: LT-146, LT-10, LT-73, LT-178, LT-160, LT-94, LT-48, LT-82, LT-86, LT-104, LT-67, LT-166, LT-114, LT-105, LT-91, LT-9, LT-127, LT-136, LT-172, LT-6, LT-93, LT-74, LT-96, LT-134, LT-72, LT-21*, LT-32*, LT-60*, LT-46*, LT-103*, LT-79*, LT-83*, LT-110*, LT-92, LT-159, LT-141, LT-111, LT-158, LT-1, LT-100, LT-57, LT-71, LT-179, LT-84, LT-115, LT-133, LT-145, LT-156, LT-80, LT-88, LT-152, LT-25, LT-26, LT-140, LT-144, LT-45, LT-30, LT-1bis, LT-161, LT-118, LT-27*, LT-125*, LT-142*, LT-121*. AMH Population from Andaman Isles, measurements taken from [33–35]. Specimens: Liang Toge, And1, And2, And3, And4, And5, And6, And7, And8, And9, And10, And11, And12, And13, And14, And15, And16, And17, And18, And19, And20, And21, And22, And23, And24, And25, And26, And27, And28, And29, And30, And31, And32, And33, And34, And35, And36, And37, And38. AMH toothless crania. PALUG Collection. Specimens: Lin112, Lin50, Lin54, Lin171, Lin149, Lin133, Lin46, Lin146, Lin74, Lin99, Lin80, Lin71, Lin45. AMH microcephalic crania. Specimens: Montefrío 32 (PALUG Collection), Mähler (cast from AIMUZ collection). AMH Pleistocene fossils. Measurements taken from [22] and [33–35]. Specimens: Predmost IV, Combe Capelle, Cro-Magnon I, Barma Grande 2, Chancelade, Obercassel 1, Obercassel 2, Abri Pataud, Cap-Blanc, Saint-Germain, Laugerie Basse N, Abri Lafaye, Grimaldi I, Grimaldi II, Mladec I, San Teodoro I, San Teodoro II, San Teodoro III, BOU-VP-16/1.

*Pan paniscus* (Zaire). RMCA collection. Specimens: 29045, 29042, 15295, 27698, 15293, 29035, 15296, 84-036M-04, 29060, 29040, 13201, 84-036M-02, 13202, 29052, 15294, 29063, 27699, 23509, 29047, 29036.

*Pan troglodytes troglodytes* (Cameroun). AIMUZ collection. Specimens: 5717, 5720, 6607, 6608, 6871, 6873, 7078, 7127, 7688, 7691, 5722, 6605, 6606, 5719, 5288, 5721, 1223, 251, 524, 1443, 6839, 7008, 312.

*Pan troglodytes schweinfurthii* (Zaire). RMCA collection. Specimens: 22925, 2298, 27697, 2488, 7004/7003, 25534, 17664, 286, 11362, 29078, 1048, 1554, 730, 15350, 11363, 7426, 9931, 4188, 5891, 5892.

*Pan troglodytes verus* (Liberia). AIMUZ collection. Specimens: 11780, 11786, 6253, 6254, 6255, 6256, 6533, 7989, 6252, 6324, 7993.

*Gorilla gorilla* (Cameroun). AIMUZ collection. Specimens: 14, 1691, 1765, 6592, 6593, 6594, 6595, 6596, 6676, 6600, 6601, 6699, 6840, 7118, 241, 917, 6603, 6602, 1648, 252, 240, 1444, 250, 760, 238, 8, 11, 6504, 6599.

*Pongo pygmaeus* (Indonesia). AIMUZ collection. Specimens: 1989, 1990, 1988, 1467, 1565, 1564, 1565, 101, 1561, 1986, 7398, 1159, 1562, 1563.

Collections studied: AIMUZ = Anthropological Institute and Museum, University of Zurich, Switzerland. RMCA = Royal Museum of Central Africa, Tervuren, Belgium. PALUG = Physical Anthropology Laboratory, University of Granada, Spain. No permits were required for the study of these specimens.

The sample of fossil hominins includes 27 individuals from four accepted genera: *Sahelanthropus*, *Australopithecus*, *Paranthropus*, and *Homo* (Table 2). Therefore, the total sample analyzed comprises 321 individuals. In order to evaluate the similarities and differences in allometric patterns, the following groups were established: (1) great apes (*Pongo*, *Gorilla* and *Pan*); (2) australopiths (*Sahelanthropus*, *Australopithecus* and *Paranthropus*); (3) extinct *Homo* (all specimens of *Homo* except *H*. *sapiens*); (4) AMH (anatomically modern humans); (5) early *Homo* (African and Caucasian *Homo* dated to the Early Pleistocene); and (6) MPEH (Middle Pleistocene *Homo*).

**Table 2:** Fossil hominins used in this study.

Specimen	Abbreviation	Taxa	Date (kyrs) / Date Reference
**TM 266-01-060-1**	TM266	*Sahelanthropus tchadensis*	7000 [41]
**KNM-WT 17000**	WT17000	*Paranthropus aethiopicus*	2520 [42]
**AL 444–2**	AL444-2	*Australopithecus afarensis*	3200 [43]
**Sts 5**	Sts5	*Australopithecus africanus*	2010 [44]
**Sts 71**	Sts71	*Australopithecus africanus*	2010 [44]
**KNM-ER 406**	ER406	*Paranthropus boisei*	1580 [42]
**OH 5**	OH5	*Paranthropus boisei*	1830 [42]
**SK 48**	SK48	*Paranthropus robustus*	1775 [45]
**DNH 7**	DNH7	*Paranthropus robustus*	1750 [46]
**KNM-ER 1470**	ER1470	*Homo rudolfensis/H*. *habilis s*.*l*.	2058 [47]
**KNM-ER 1813**	ER1813	*Homo habilis*	1650 [48]
**OH 24**	OH24	*Homo habilis*	1800 [49]
**D 3444**	D3444	*Homo georgicus/H*. *habilis s*.*l*.*/H*. *erectus s*.*l*.	1815 [50]
**D 2700**	D2700	*Homo georgicus/H*. *habilis s*.*l*. */H*. *erectus s*.*l*.	1815 [50]
**D 2282**	D2282	*Homo georgicus/H*. *habilis s*.*l*. */H*. *erectus s*.*l*.	1815 [50]
**D 4500**	D4500	*Homo georgicus/H*. *habilis s*.*l*. */H*. *erectus s*.*l*.	1815 [50]
**KNM-ER 3733**	ER3733	*Homo ergaster/H*. *erectus s*.*l*.	1630 [51]
**KNM-WT 15000**	WT15000	*Homo ergaster/H*. *erectus s*.*l*.	1500 [52]
**Stw 53**	Stw53	*Homo gautengensis/H*. *habilis s*.*l*.	1650 [53]
**Sangiran 17**	Sang17	*Homo erectus*	800 [52]
**Kabwe**	Kabwe	*Homo rhodesiensis/H*. *erectus s*.*l*.	300 [52]
**SH Cranium 5**	SH5	*Homo heidelbergensis*	350 [54]
**Steinheim**	Steinh	*Homo heidelbergensis*	250 [55]
**Petralona**	Petr	*Homo heidelbergensis*	252.5 [56]
**Shanidar I**	Shan1	*Homo neanderthalensis*	100 [52]
**La Chapelle**	LaCh	*Homo neanderthalensis*	52 [52]
**La Ferrasie I**	LaFerr1	*Homo neanderthalensis*	72 [52]
**LB-1**	LB1	*Homo floresiensis*	18 [57]

*Homo georgicus/H*. *habilis s*.*l*.*/H*. *erectus s*.*l*. refers to the Dmanisi paleodeme.

**Fig 1:**
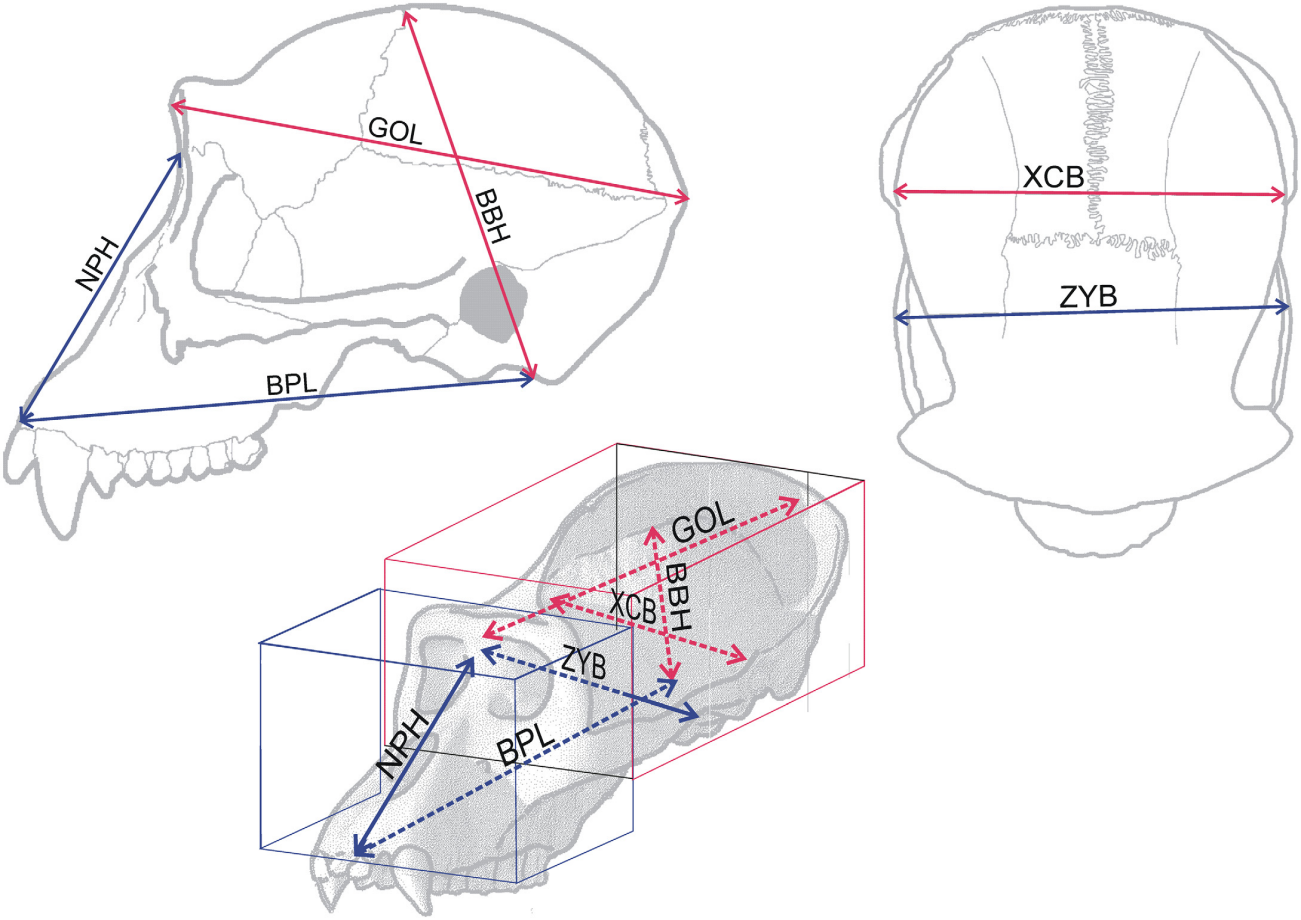
Craneometric variables used in this study. For abbreviations, see text. BBH was estimated following [58]; in those specimens with sagittal crest, bregma was placed in the plane surrounding the cranial vault surface. BPL was estimated in the toothless specimens placing prosthion on the middle line of the skull, at the most inferior point of the maxillo-alveolar process. Note that the three variables of each cranial module are linearly independent, as each of them cannot be obtained as a linear combination of the other two.

Six metric variables that allow characterizing the overall dimensions of each cranial complex were chosen. Three belong to the neurocranium: glabella-opistocranion length (GOL), basion-bregma height (BBH) and maximum biparietal cranial breadth (XCB). The other three were measured in the splanchnocranium: basion-prosthion length (BPL), nasion-prosthion height (NPH) and bizygomatic breadth (ZYB) (Fig 1).

The three variables of each cranial module are contained in orthogonal planes. This means that they are *a priori* linearly independent, which entails the size of each module to be approached by its length, width and height. Given that the variables selected are standard paleoanthropological measurements, they were mostly collected from the literature (S1 Table).

However, in a few cases the measurements were not available in the bibliography and were measured on casts (4 individuals, 6.2% of the measurements taken in fossils), virtual reconstructions (1 individual, 1.2% of the measurements), and photographs (6 individuals, 10.5% of the measurements). When the zygomatic arches were partially absent, they were reconstructed conservatively by joining the preserved part of the zygomatic process of the temporal bone with the zygomatic bone. If one zygomatic arch was lost, ZYB was estimated with standard photographic software using the mirror image of the preserved side. The measurements taken on photographs were measured independently by two of us and averaged (in any case, the discrepancies were always less than 2%). All the variables were transformed logarithmically prior to subsequent statistical analyses.

Data representativeness was tested by a comparison of our sample of anatomically modern humans with Howells craniometric dataset, which includes measurements from 2,524 human crania from 28 populations (S1 Text). In this comparison, the test of Lubischew was used for estimating the degree of overlap between both distributions for each variable (S2 Table). In addition, a principal components analysis was performed over the values of the log-transformed craneometric variables joining our population with Howells dataset, in order to evaluate the patterns of morphospace occupation by both samples (S3 Table, S1 Fig). The results obtained indicated that, compared with Howells dataset, our sample of human crania is not biased (S1 Text).

The covariation between both cranial modules was studied by means of the two-block partial least squares technique (2B-PLS) [59], an approach used previously for studying the morphological integration of the cranium (e.g., [2, 8,16, 60]). The correlation matrix to be decomposed was obtained from the specimens of the living species. Assuming that most of the variation and covariation in shape is due to the mean species differences, the first dimension of PLS should describe a pattern of evolutionary integration [8]. In order to assess developmental integration, a pooled within-species 2B-PLS analysis was also performed subtracting the differences in species means to the previous data.

Given that the cranial modules can be correlated indirectly via their correlation with cranial size, the two previous analyses were also accomplished by dividing each row (specimens) by the geometric mean of its six variables [61,62]. After this size standardization, every specimen has a geometric mean of 1. This method can be conceived as an equivalent of the "simultaneous-fit" approach (*sensu* [63]) for metric variables, because each variable is scaled to the size of the whole structure. Finally, using the previous results as a framework, a PLS analysis was also carried out including the fossils of adult specimens.

Factor analysis was also used for analyzing the pattern of covariation. This approach has been applied in the context of morphological integration (e.g., [64,65]), as in the case of the previous method. However, a factor analysis does not allow identifying modules exclusively from morphometric measurements [2]. As indicated above, we assume on the basis of previous studies that the face and the neurocranium are the two most prominent cranial modules. In fact, the own recognition of the modular nature of the cranium implies that each of its two modules can vary with certain independence from the other. In consequence, the pattern of covariation among the selected variables should reflect such modularity, at least to a certain extent. Factor analysis has been widely used for analyzing allometries from a multivariate point of view [66]. This technical approach is specifically robust for the search of general patterns, because a small error in the estimation of the variables for a given individual results only in a minimal change in its position within the multivariate space. For example, two new sets of anthropological measurements were recently published for cranium OH5 (*P*. *boisei*) and ER 1813 (*H*. *habilis*) based on two virtual reconstructions [67,68] that differ somewhat from those published previously [58]. For this reason, we used two approaches for testing the robustness of our analyses over the cranial specimens of extinct hominins. First, in those cases in which several measurements were available for a given specimen (e.g., OH5, ER 1813, SK48, Stw53 and Sts5; see S4 Table), these were employed as independent case studies for evaluating the consistency of their scores on the principal components. Results obtained (S2 Fig) showed that the projections for the same specimen were always in close proximity. The second approach used 500 simulations for each fossil cranium in which the original measurements were varied at random. In all cases, their projections plotted in close proximity to our data (S3 Fig).

In order to characterize independently changes in size and shape (i.e., allometries), we followed the two major conceptual frameworks of allometry [66], the Huxley-Jolicoeur school, which proposed the use of the principal component of the log-transformed variables that can be interpreted in an *ad hoc* manner as a size vector, and the Gould-Mosimann school, which used the geometric mean of all variables as a size estimator (S1 Text). The latter approach allowed to calculate the direction of isometric change as a straight line at equal angles to all coordinate axes in the morphospace of log-transformed traits (i.e., the vector that is a scalar multiple of [1, 1, 1, …, 1]) [66]. The scalar product of this vector with the principal components provides the angle that separates them.

All the slopes presented here correspond to regressions adjusted with the reduced major axis method and have been performed using the free-downloaded program "PAST", implemented by [69]. Statistical significance of the slopes and correlations was tested with permutation tests, using 10,000 replicates. Null hypotheses of equality of slopes were tested following [70].

## Results

For clarity purposes, the results of factor analysis are presented first. The two first factors obtained account for 61.2% and 32.5% of the original variance, respectively. Factor loadings of the variables measured in the neurocranium are positive in the first factor and those for the splachnocranium are negative (Table 3).

**Table 3:** Summary of factor analysis.

Variable	h^2^	1^st^ Factor	2^nd^ Factor
**logGOL**	0.932	0.651	0.712
**logBBH**	0.938	0.887	0.388
**logXCB**	0.948	0.914	0.336
**logNPH**	0.920	-0.821	0.497
**logBPL**	0.956	-0.894	0.396
**logZYB**	0.927	-0.406	0.873
**Eigenvalue**		3.684	1.937
**Variance (%)**		61.41	32.28
**Cumulative var. (%)**		61.41	93.69

The first column shows the communalities of each variable retained by the two first factors (h^2^). The second and third columns show the factor loadings of each variable on the first and second factor, respectively. The eigenvalues of both factors and the percentages of the total variance that they account for are also provided.

**Fig 2:**
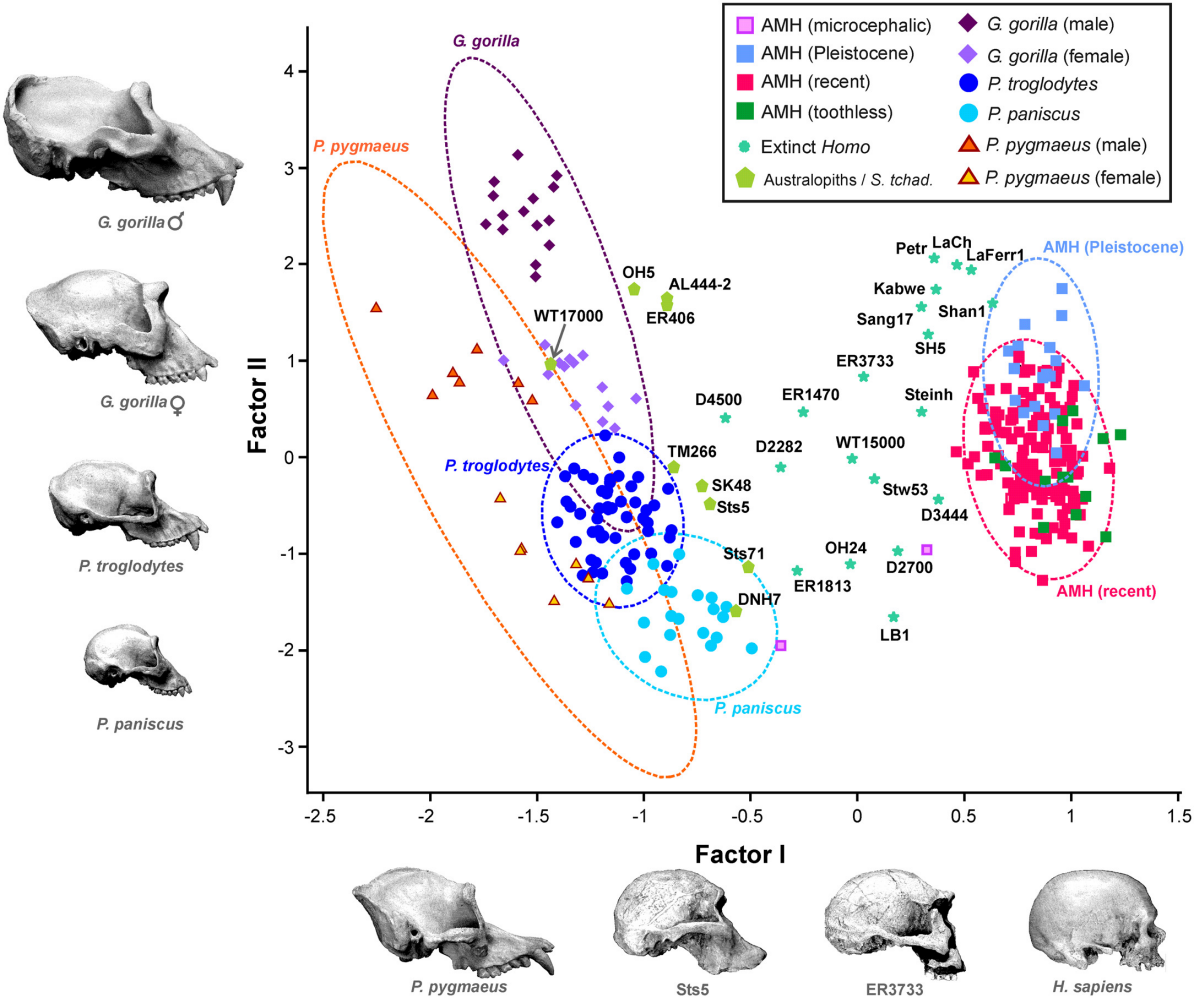
Bivariate plot of the scores of the specimens analyzed on the first two factors. Dotted lines enclose the 95% confidence ellipses for the living species.

Basically, this allowed us to interpret the first factor as a shape axis and showed that the main source of variation within the dataset is associated with an inverse relationship between the sizes of both cranial complexes (i.e., the sample varies more in shape than in size). Consequently, the individuals with the largest faces and smallest neurocrania (i.e., orang-utans) score negatively and are projected on the left side of this axis, while those individuals that show the opposite condition (AMH) score positively on the right side (Fig 2).

**Fig 3:**
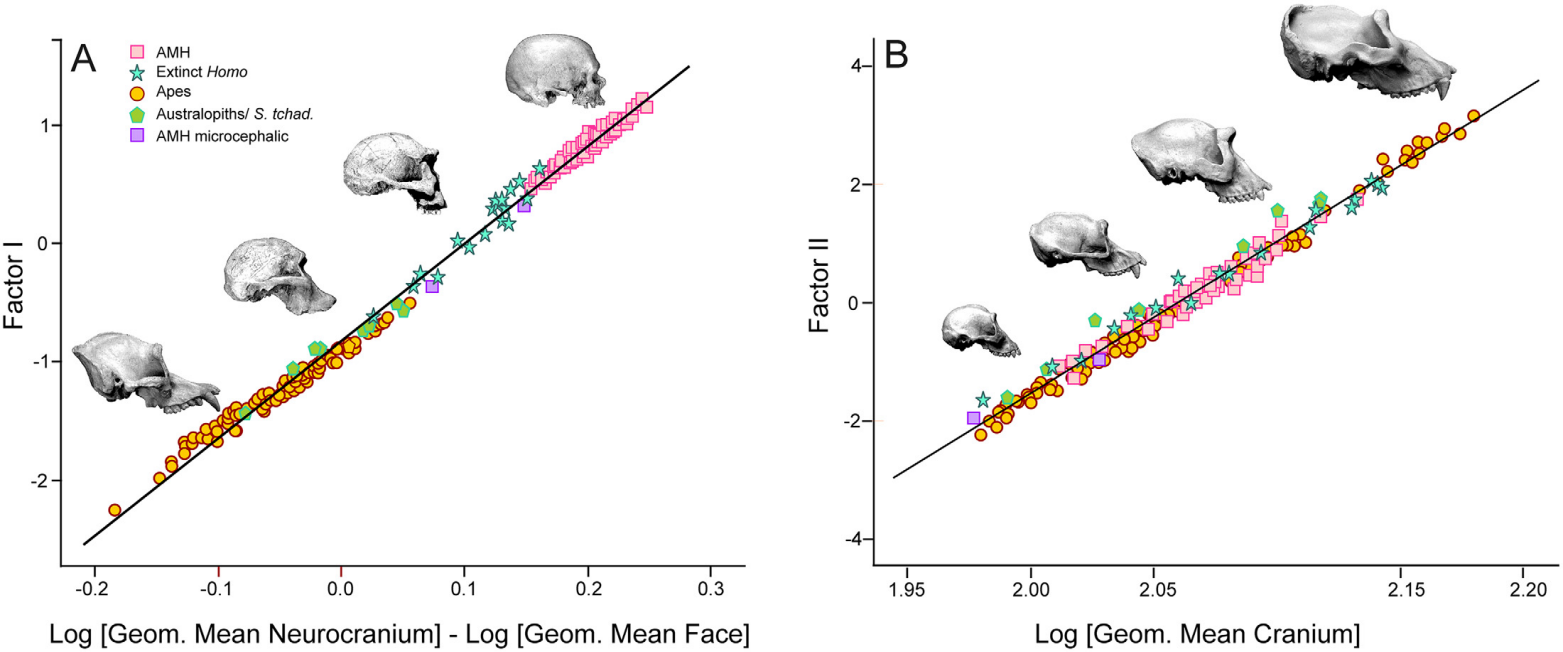
Plots of the specimens’ scores for Factors I and II on their geometric means. A) Bivariate plot of FI scores on the difference between the log_10_-transformed geometric mean for the three neurocranial variables and the corresponding value for the three facial variables. B) Bivariate plot of FII scores on the log_10_-transformed geometric mean for the six analyzed variables.

There is also a close correspondence between the scores on this axis and the logarithm of the quotient between the geometric means of the variables measured in the face and in the neurocranium (*r* = 0.998; *p* < 10^−300^) (Fig 3A). Therefore, this factor reflects the basic pattern of morphological integration between the neurocranium and the face in hominoids. However, bizygomatic width does not correlate to the same degree than the other five variables with this factor, as noted by a lower factor loading value (Table 3). This results in part from the fact that this measurement has a relatively high ratio of intraspecific/interspecific variance.

A comparison of the results of our factor analysis with those obtained using geometric morphometric methods with three-dimensional landmarks [13] is provided in S1 Text and S4 Fig.

The second factor can be interpreted as a size axis, because all metric variables show positive loadings on it (Table 3). Such interpretation is supported by the high positive correlation that this axis shows with the geometric mean of the six variables used in the analysis (R^2^ = 0.992; *p* < 10^−284^) (Fig 3B). The smallest crania (i.e., bonobos, microcephalic modern humans, DNH 7 and LB-1) have the lowest scores on this axis, while the largest crania (gorilla males) show the highest ones (Fig 2). In addition, the vectors that connect within the morphospace isometric organisms are positioned at an angle of 4.56° with respect to the second factor (and thus, at 85.43° with the first axis).

All these results reinforce the interpretation that the first factor essentially measures shape changes, while the second one is basically a size vector.

As a general rule, the females of the highly dimorphic species (e.g., orang-utan and gorilla) show faces less developed in relation to the neurocranium than the males. Also, the edentulous modern that have an advanced degree of alveolar resorption show faces that are slightly smaller than those of toothed individuals. Finally, the two microcephalic AMH analyzed score distantly. Although both have a very small cranium, this is coherent with the heterogeneous nature of the teratologies that lead to human microcephaly [71].

**Fig 4:**
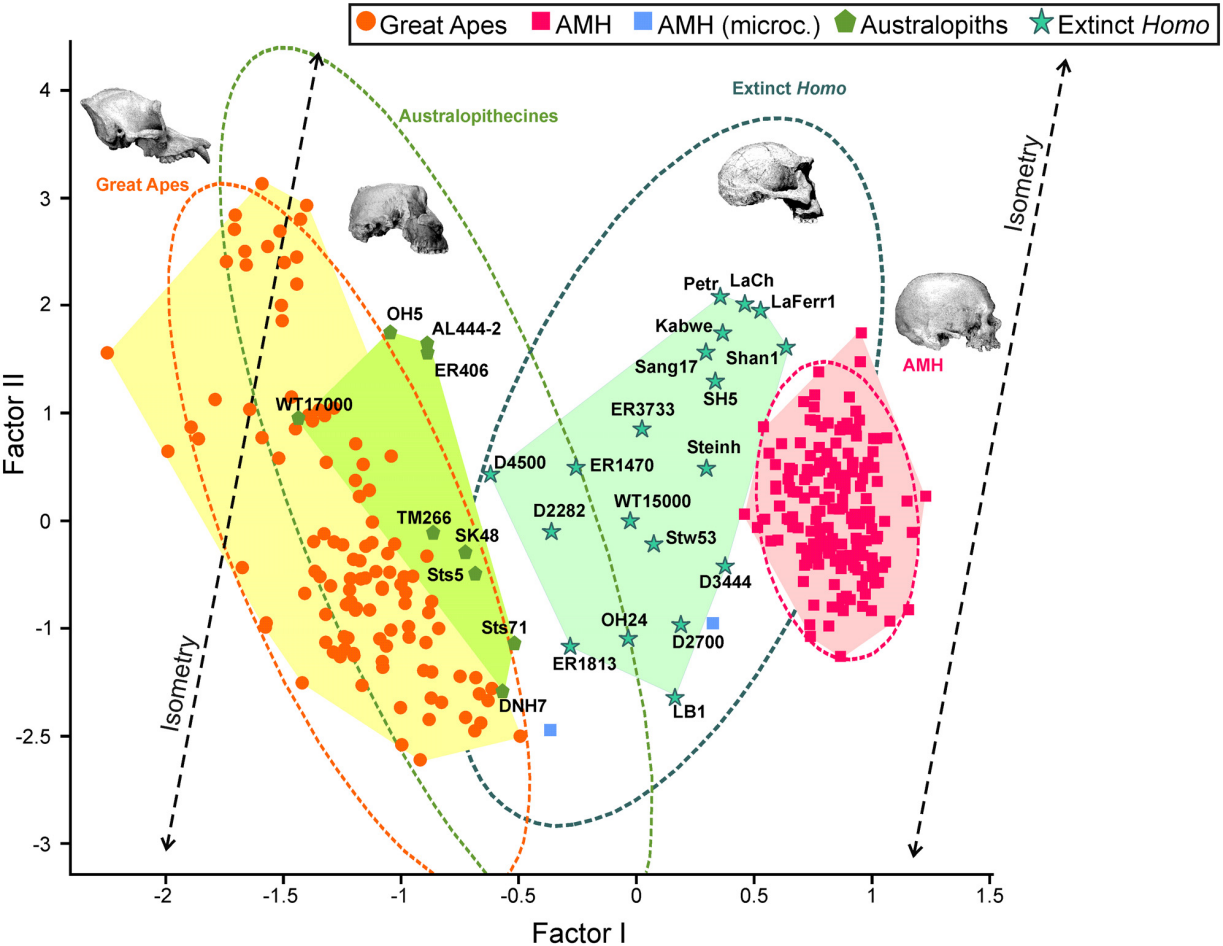
Bivariate plot of the scores for the specimens analyzed on the first two factors. Dotted lines enclose the 95% confidence ellipses for the four hominoid groups considered in this study.

There is a well-defined gap between the great apes and the modern humans in the cranial morphospace, and this region is occupied by most fossil hominins (Figs 2 and 4). As a general rule and with the only exception of the robust australopiths, which are contemporary to early *Homo*, the older a hominin is, the more ape-like it resembles in the face-neurocranium relationship (i.e., it scores more negatively in the first factor). ‘ 266-01-060-1 scores very close to the common chimpanzees. The Eastern African australopiths constitute a relatively homogenous group and are positioned in size between the males and females of gorilla. The Southern African australopiths show similar scores on the shape vector, but have lower projections on the size vector. The most plesiomorph australopith is WT17000, whose face-neurocranium ratio is typical of a great ape.

The specimens of early *Homo* plot on the region of the morphospace situated between the australopiths and AMH. The Dmanisi population, the one that dispersed first out of Africa, shows more morphological disparity than the African specimens of *H*. *habilis*, *H*. *rudolfensis* and *H*. *ergaster*. This may result in part from the inclusion in the analysis of edentulous cranium D344 [28], as toothless crania from AMH are also slightly displaced to more positive scores on the first factor compared to toothed individuals, and also from the inclusion of the very robust cranium D4500 [29], which shows the combination of a small braincase and a very prognathic face. The crania of Middle Pleistocene *Homo* (*H*. *erectus*, *H*. *rhodesiensis* and *H*. *heidelbergensis*) represent a very homogeneous group in the shape component, lying close to *H*. *neanderthalensis*. Pleistocene and recent AMH show identical scores in the first axis, although the former are slightly displaced to more positive values in the second one, which denotes their larger size. Finally, LB-1 has the lowest score on the size vector among the fossil hominins, scoring on the shape axis between the earliest *Homo* and Middle Pleistocene *Homo*, close to Sangiran 17, the only cranium of *H*. *erectus* that preserves its face. LB-1 scores in shape close to Montefrío32, a microcephalic modern human, but D2700 is more closely positioned to this pathologic cranium.

Allometric growth patterns within species (or groups) were characterized using the reduced major axis regressions of the first factor on the second (Table 4). Most living species show negative allometries. In other words, for a given group, the larger a cranium is, the smaller its neurocranium is compared to its face. To a large extent, this may be the consequence of sexual dimorphism. In which concerns intertaxonic allometries, the adults of the African apes line relatively well within a common pattern, as in the case of the australopiths (particularly, if WT17000 is excluded), and both show more or less parallel lines (Fig 2). On the contrary, the group “extinct *Homo”* is the only one that shows positive allometry. Similar results were obtained using the geometric mean as a size estimator (S1 Text, S5 and S6 Tables).

**Table 4:** Reduced major axis regressions in different groups of hominids for the scores of the specimens in the first factor (shape vector) on the scores in the second factor (size vector).

Group	N	R^2^	Slope	Bstr95% Slope	*p* (r = 0)
**AMH**	174	0.024	-0.247	[-0.286; -0.208]	0.0418
***Pan paniscus***	20	0.040	-0.493	[-1.583; -0.255]	n.s.
***Pan troglodytes***	54	0.011	-0.370	[-1.161; -0.267]	n.s.
***Gorilla gorilla***	29	0.553	-0.197	[-0.237; -0.155]	<0.00001
***G*. *gorilla* ♂**	15	0.016	-0.327	[-1.174; -0.167]	n.s.
***G*. *gorilla* ♀**	14	0.487	-0.574	[-0.809; -0.273]	0.0055
***Pongo pygmaeus***	14	0.709	-0.274	[-0.360; -0.217]	0.0002
***P*. *pygmaeus* ♂**	7	0.490	-0.725	[-2.509; 0.425]	n.s.
***P*. *pygmaeus* ♀**	7	0.664	-0.505	[-0.720; -0.145]	0.0255
**Great Apes**	117	0.512	-0.233	[-0.269; -0.1912]	<0.00001
**African apes**	103	0.668	-0.195	[-0.218; -0.168]	<0.00001
**Australopiths**	9	0.500	-0.220	[-0.337; -0.055]	0.0331
Australopiths*	8	0.831	-0.137	[-0.178; -0.082]	0.0016
**Extinct *Homo***	19	0.259	0.277	[0.148; 0.386]	0.0260

R^2^: coefficient of determination; *p*: probability r = 0; n.s.: non significant (*p >* 0.05). Bstr95%: bootstrapped 95% confidence intervals (2,000 replicates). Australopiths* refers to all australopith crania except WT17000.

All 2B-PLS analyses performed yielded essentially a one-dimensional solution (Table 5) and, consequently, subsequent comments will refer only to this dimension. Both the non-pooled and pooled within species 2B-PLS analyses for the living hominoids using standardized variables (Table 5, columns A and B for patterns of evolutionary and ontogenetic integration, respectively) showed positive and negative loadings for the variables measured in the neurocranium and the splachnocranium, respectively. The correlations between both blocks of variables are positive, which indicates that an increase in the size of one cranial module is associated with a parallel decrease in the other.

**Table 5:** Summary of the 2B-PLS analyses between the variables taken in the neurocranium and the splachnocranium for adult hominoids.

Variable	A	B	C
**logGOL**	0.552	0.308	0.547
**logBBH**	0.595	0.682	0.597
**logXCB**	0.585	0.664	0.587
**logNPH**	-0.608	-0.761	-0.613
**logBPL**	-0.622	-0.576	-0.627
**logZYB**	-0.494	-0.298	-0.480
**Singular value**	2.594	0.899	2.540
**% Variance**	99.99	97.69	99.99
**Correlation**	0.992	0.961	0.992

See [59] for details on the statistics. All the correlations are significant at *p <* 0.001. Non-pooled (column A and C) and pooled within species (column B) size-standardized variables were obtained dividing the craniometric measurements by their geometric mean. The analyses for columns A and B were performed only with the living species, while column C includes also extinct taxa.

**Fig 5:**
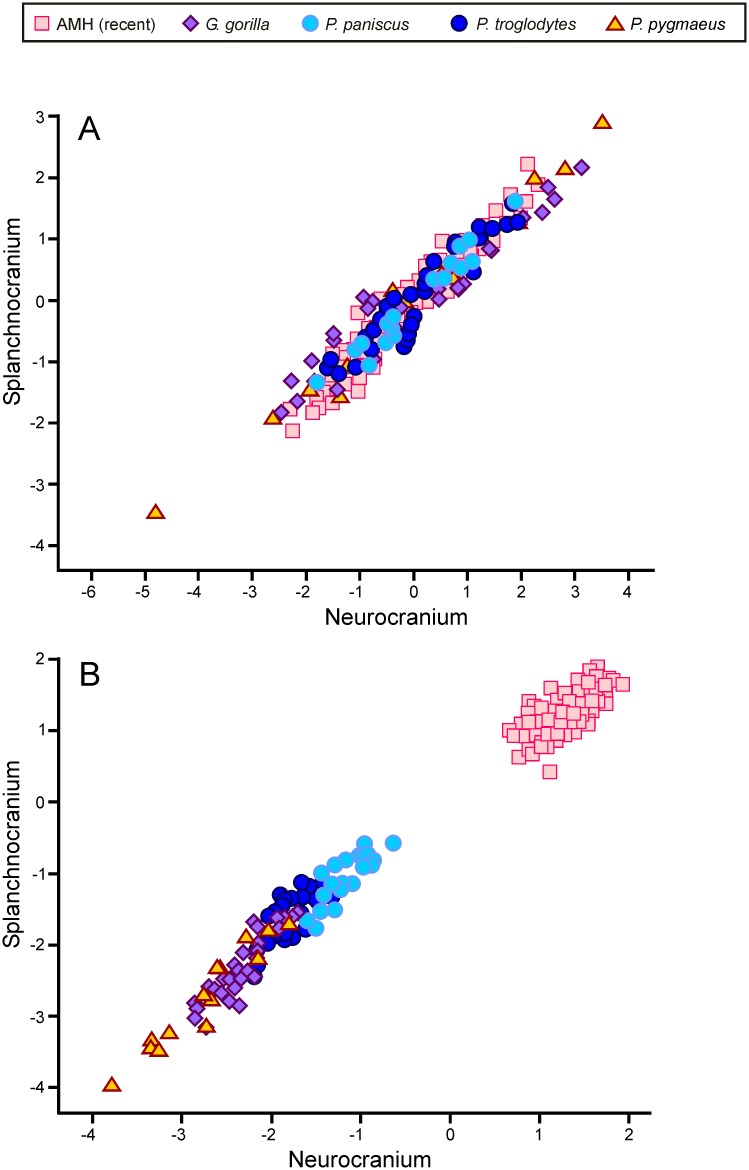
Two-block partial least squares plots. Non-pooled (A) and pooled within species (B) 2B-PLS plots of the face *vs*. the neurocranium for the living species, respectively. Non-pooled (C) and pooled within-species (D) 2B-PLS plots of the face *vs*. the neurocranium for size-scaled living species, respectively. The correlations between the scores on each block are significant for all species (*p* < 0.05).

**Fig 6:**
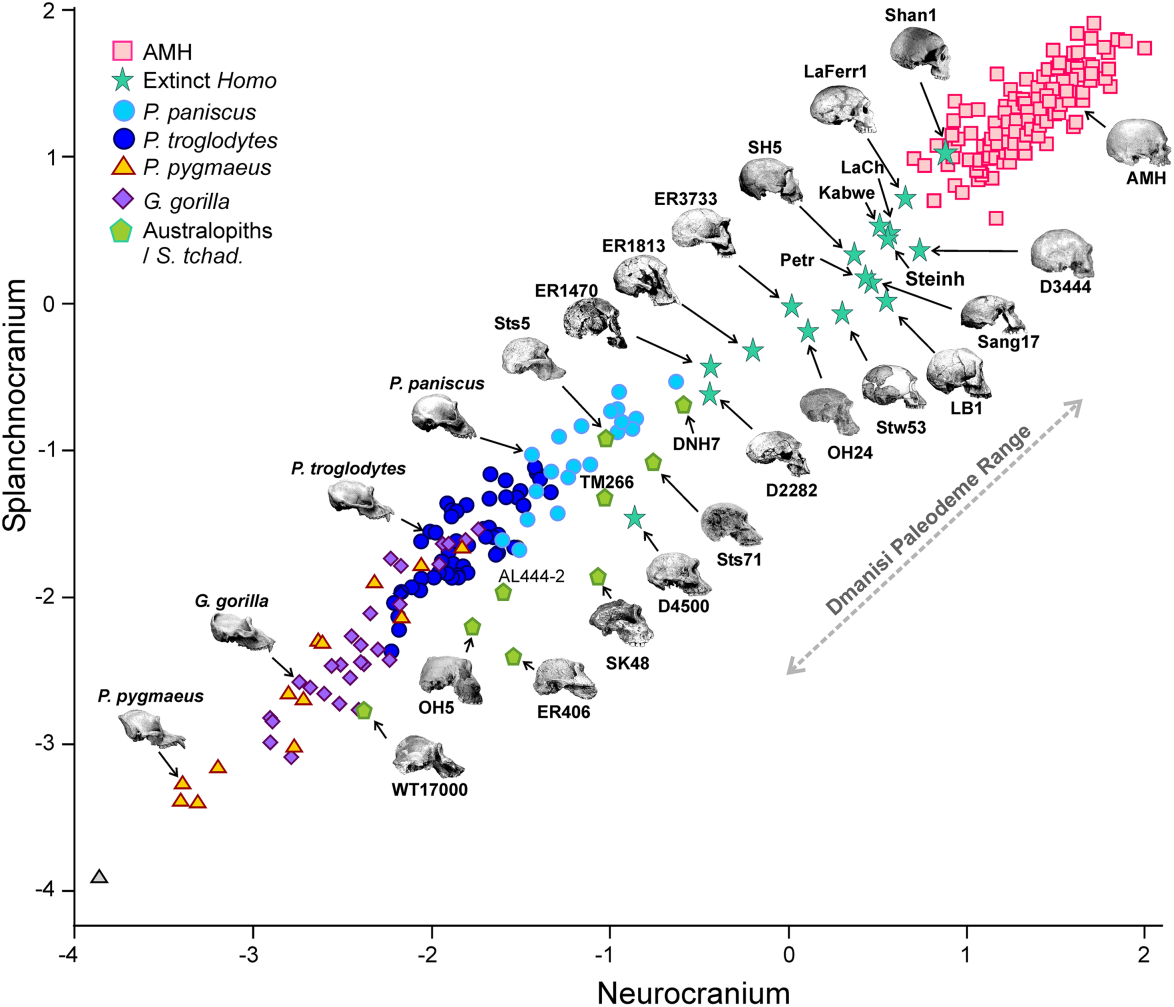
Non-pooled within-species 2B-PLS plots of the face *vs*. neurocranium for size-scaled adults of the living and extinct hominoid species. The correlations between the scores on each block are significant for all species/groups (*p* < 0.05).

The plot for the analysis non-pooled within species shows that the living hominoids line in a well-defined sequence: *P*. *pygmaeus*, *G*. *gorilla*, *P*. *troglodytes*, *P*. *paniscus* and, distant from them, AMH (Fig 5B). All species show significant correlations between their scores on both dimensions, which is even more obvious in the pooled within-species plot (Fig 5A, column B in Table 5). In conclusion, ontogenetic and evolutionary integration run in the same direction (i.e., the relative sizes of the splachnocranium and the neurocranium relate inversely both within and between species). The inclusion of fossil hominins results in similar loading coefficients for the 2B-PLS analysis (compare Table 5, columns A with C, and Fig 5A with Fig 6) and does not change the pattern of evolutionary integration depicted by the living hominoids.

The australopiths show a wide range of values (Fig 6), which is coherent with the multispecific nature of a group that includes up to six different species, and most of them line in parallel to the great apes. In general terms, the crania with a relatively larger face (e.g., *P*. *aethiopicus*, *P*. *boisei* and *A*. *afarensis*) are closer to *P*. *pygmaeus*, while those with a more developed neurocranium (e.g., *A*. *africanus*, *P*. *robustus* and *S*. *tchadensis*) are closer to *P*. *paniscus*. If D4500 is excluded, no australopith reaches the lowest neurocranial dimensions of extinct *Homo* (Fig 6), which fill the gap between the australopiths and AMH following a more or less linear trend.

**Fig 7:**
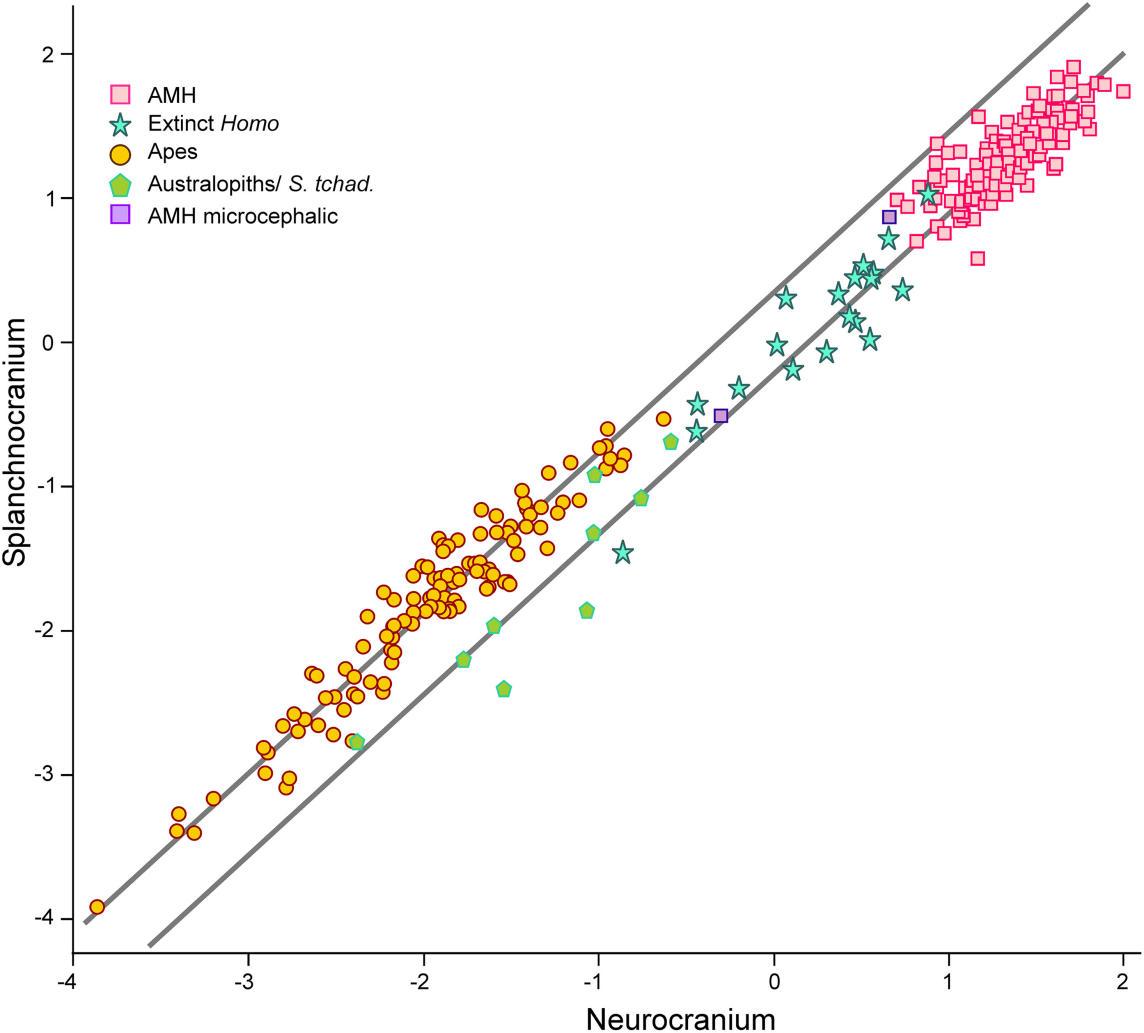
Non-pooled within-species 2B-PLS plots of the face *vs*. neurocranium for scaled specimens. The correlations between the scores on each block are significant for all species/groups with the exclusion of microcephalic crania (*p* < 0.05).

As in the case of the living species, the groups “australopiths” and “extinct *Homo”* have significant correlations between their scores on both PLS's. However, australopiths, extinct *Homo* and AMH seem to line in parallel to the great apes (Fig 7). The RMA slopes for both sets are quite similar (1.119 and 1.112 for hominins and great apes, respectively). This suggest that although the pattern of covariation between their cranial modules is basically the same, a great ape couldn’t reach the morphology of an AMH simply by increasing the size of its neurocranium. In addition, the australopiths do not show a correlation between the geological age of the specimens and their projection onto this line (both including and excluding *S*. *tchadensis*). In contrast, extinct *Homo* shows a very significant correlation between both variables (r = -0.730; *p* < 8.9 10^−4^), which determines a clear evolutionary trend in the genus. However, it is debatable to which extent such trend is cladogenetic or anagenetic.

## Discussion

Our results show that the use of a relatively low number of anthropometric measurements allows characterizing the patterns of covariation between the overall dimensions of the neurocranium and the splachnocranium. Moreover, these standard variables can be measured in many fossil crania, which allows increasing the sample of hominins that can be analyzed simultaneously. The general patterns described here are consistent with others published using more accurate approaches, as those based on geometric morphometrics and developed on less complete datasets [13,72]. There are several reasons for this correspondence. One is that standard metric variables are inter-landmark distances and thus correlate to some extent with their shape coordinates. In addition, the modular nature of the cranium implies that a change in a given trait will lead to changes in other traits. For example, the variation described by the first principal component of [13] was mainly related to changes in the relative sizes of the neurocranium and the splachnocranium.

As pointed out by [2], the existence of cranial modules cannot be reliably identified from analyses of phenotypic covariance in non-experimental data. However, once the modules have been identified, the analyses of covariation matrices can help in estimating patterns of integration. Our factor analysis describes adequately the major patterns of evolutionary integration, because most of the variation and covariation in the cranial shape of hominoids results from differences between the species means [8]. In which concerns evolutionary integration, the information provided by factor analysis and 2B-PLS was essentially the same, but the former is better for describing the allometries between both cranial complexes. However, the appropriate 2B-PLS analysis for assessing developmental integration must be based on the pooled within-species covariance matrix, as indicated by [8].

Our results show a similarity in the overall pattern of developmental integration of the cranium for humans and the great apes, in agreement with previous studies (e.g., [8,16]). For this reason, it is not unreasonable to assume that the extinct hominins shared with the modern taxa the same developmental program. However, the use with extinct taxa of covariance patterns deduced from extant species (e.g., [16, 73]) introduces a cautionary note. The inverse correlation between the relative dimensions of the two cranial modules in the analyses within and between species can denote the existence of a developmental constrain, thus limiting the number of evolutionary paths on which natural selection could act. As pointed out by Alberch [74], the externalism vs. internalism debate showed that evolution is the outcome of developmental dynamics and selective factors. Similarly, Mitteroecker and Bookstein [8] indicated that evolutionary integration is a consequence of developmental integration and coinheritance in the context of selective regimes.

**Fig 8:**
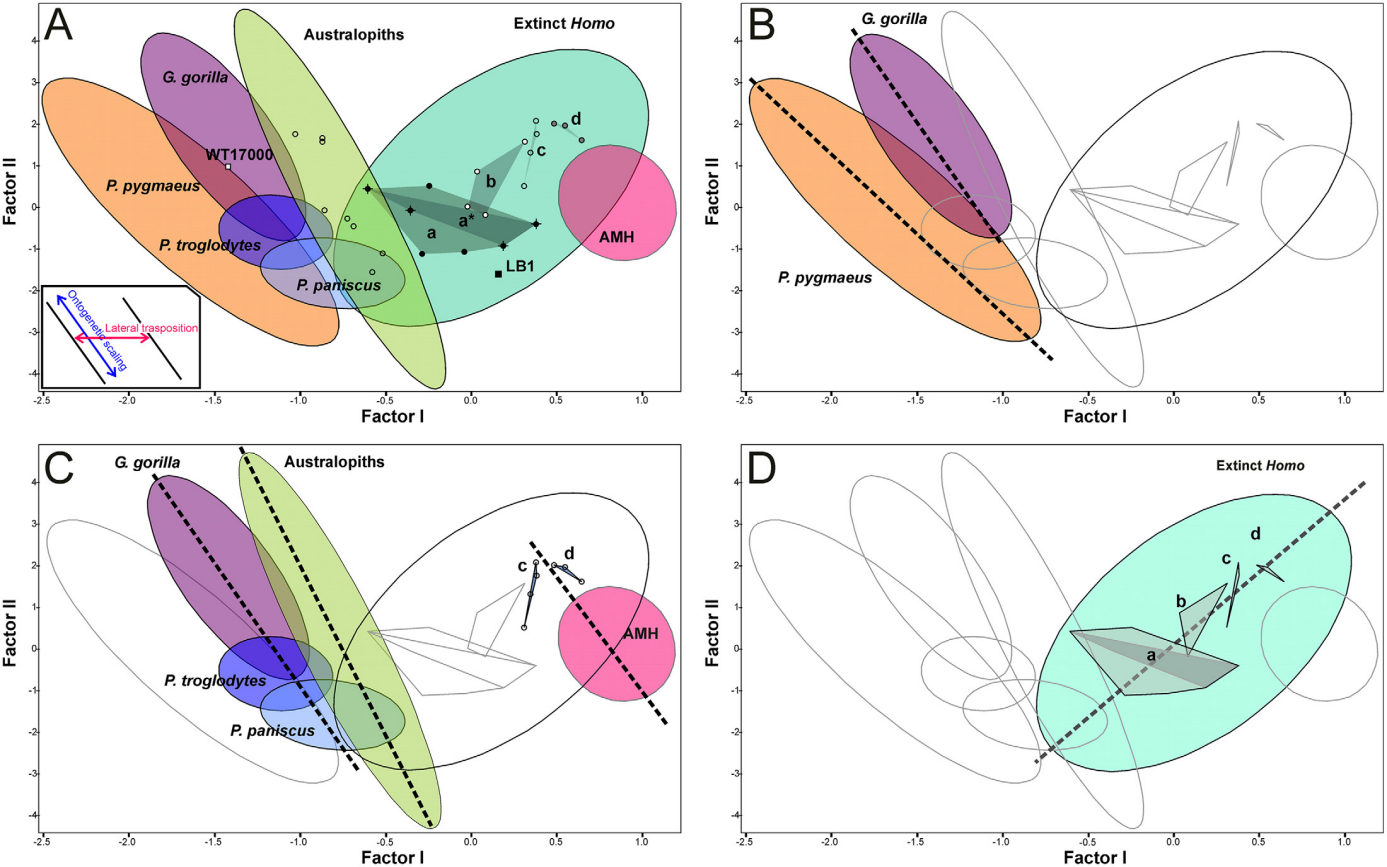
Bivariate plots of the scores for different taxonomic sets on the first two factors. A) Bivariate plots of FII on FI scores. Ellipses enclose the 95% confidence regions. The ellipse for australopiths was plotted excluding WT-170000; a: convex hull for habilines; a*: Dmanisi paleodeme; b: convex hull for erectines; c: convex hull for *H*. *heidelgergensis*; d: convex hull for *H*. *neanderthalensis*. B) Intraspecific allometries resulting from sexual dimorphism. C) Interspecific allometries that run in parallel to ontogenetic scaling. Note that LB1 relates with the habilines through ontogenetic scaling. D) Interspecific allometry opposed to ontogenetic scaling.

The combined patterns of developmental and evolutionary integration define a set of allometric trends, which describe how the two main cranial modules can change their relative sizes with overall cranial size (Fig 8). These allometries can be grouped into three categories: (1) intraspecific variation due to sexual dimorphism, as those exhibited by *P*. *pygmaeus* and *G*. *gorilla* (Fig 8B); (2) interspecific variation resulting from ontogenetic scaling (Fig 8C), as those depicted by the sequence *P*. *paniscus*-*P*. *troglodytes*-*G*. *gorilla*, the australopiths, and the sequence AMH-latest extinct *Homo* (groups c and d in Fig 8C), sequences that are laterally transposed; and (3) interspecific variation among extinct *Homo* that probably results from lateral transposition as a consequence of having different mean sizes for both cranial complexes (Fig 8D). Obviously, these allometric trends do not necessarily imply the existence of ancestor-descendant relationships or phylogenetic proximity between the taxa studied.

Those sets with allometric rules that follow ontogenetic polarity (1 and 2) could acquire more ape-like proportions simply by increasing cranial size: for example, the females of the highly dimorphic apes have smaller crania and are more human-like than the males; bonobos are less ape-like than gorillas because they are smaller. Other way to achieve this is by lateral transposition: australopiths are less ape-like than gorillas and chimps because they follow allometric rules with a different basal proportion (i.e., Y-intercept) between both cranial complexes. The only exception is the group “extinct *Homo”*, in which the allometric trend is the opposite of the one that results from ontogenetic scaling. This indicates a basic difference between the australopiths and the genus *Homo*. All australopiths can be considered as ontogenetic scaled versions of the same organism, as suggested by [75]. On the contrary, evolution within the genus *Homo* was mainly based on lateral transpositions, which changed the ape-like, plesiomorph configuration of the relative dimensions of the neurocranium and the face according to the increase in cranial size. However, the cranium WT-17000 is an exception to this general rule (Fig 8A), as it departs from the australopith allometry to enter well into the region of the morphospace occupied by the living great apes. This was presumably achieved by lateral transposition, as we must assume that its ancestor (a species close to *A*. *afarensis*) followed the developmental logic of australopiths. In this case, it is not unreasonable to infer a change of adaptive zone for this species (*sensu* [76]).

The case of LB1 is also very interesting. If we assume that the overall pattern of developmental integration of hominins is conserved, ontogenetic polarity could be determined. This makes possible to connect allometry with heterochrony [66]. It is obvious that *H*. *floresiensis* does not follow the allometric trend of Middle Pleistocene *Homo*, neantherthals and AMH. If *H*. *floresiensis* derived from AMH in insular conditions, it would have been through lateral transpositions. This means that *H*. *floresiensis* should be paedomorphic *s*.*l*. in size and peramorphic *s*.*l*. in shape with respect to AMH. However, it is easier to connect LB1 with the habilines by ontogenetic scaling (Fig 8A), which means that LB1 would be paedomorphic in both size and shape. This is a more parsimonious view, as it agrees with interpretations of *H*. *floresiensis* as a dwarfed early *Homo* (e.g., [72,77]).

There is another important difference between the australopiths and the genus *Homo*: the evolutionary allometry (*sensu* [66], not [78]) depicted by the australopiths is timeless, while in *Homo* it defines a clear evolutionary trend. In the case of *Homo*, this implies that two contemporary crania can differ in shape if they also differ in size; an extreme example of this would be the Dmanisi paleodeme.

As pointed out by [6], much of the diversity in primate cranial morphology is tied to the relative importance of those skull regions that are involved in different functions (e.g., the brain, the sensory organs and the masticatory complex). Given that the facial component houses the sensory organs and an important part of the masticatory complex, whereas the neurocranium encases the brain, it might be reasonable to assume that the differences in the relative size of both cranial modules plus the differences in overall skull size would define differences in adaptive zones. If this were so, all the australopith species would have occupied a similar adaptive zone, characterized by a predominance of the facial component. In contrast, the genus *Homo* changed consistently its adaptive zone since its own origin, which was achieved by increasing the neurocranial module at the expense of the face.

The sustained trend of encephalization that took place during the evolution of the genus *Homo* resulted in an increase of the energetic cost of maintenance for an expanded brain, which in modern humans represents nearly one quarter of the basal metabolic rate. According to the “expensive tissue” hypothesis [79], the increase in brain size was closely tied to a parallel decrease in gut size, the only way of compensating the increasing metabolic demands of the brain. This ultimately resulted in a reduction of the relative dimensions of the face and teeth, which represented an additional metabolic saving [80] and probably forced these hominins to adopt a more carnivorous diet. The appearance of the first stone tools, dated ~2.5 Myr ago [81] and coincident with the appearance of the genus *Homo*, made possible a more effective access to the carcasses of ungulate prey partially consumed by the large hypercarnivores [82]. This could enhance efficiency in the obtaining of high quality resources such us meat and fat through confrontational scavenging [83–87]. In addition, a change towards more elaborated social relationships would have contributed to optimize the obtaining of animal resources, as evidenced in the Early Pleistocene sites of southern Spain [86], with the consequent selective advantage [28, 87, 88].

## Supporting Information

S1 Fig Principal components analysis of craniometric variation in the two samples compared (Howells dataset and this study). (DOCX)

S2 Fig Morphospace projections of alternative craniometric measurements. (DOCX)

S3 Fig Morphospace projections of simulated craniometric measurements. (DOCX)

S4 Fig Comparison between the principal components of Guy et al. and the factor analysis of this study. (DOCX)

S1 Table Measurements of fossil hominin crania. (DOCX)

S2 Table Comparison with Howells dataset. (DOCX)

S3 Table Principal components analysis of craniometric variables in our sample of *Homo sapiens* and Howells dataset. (DOCX)

S4 Table Measurements for several hominin crania from different sources. (DOCX)

S5 Table Analysis of allometric change in hominoids. (DOCX)

S6 Table Comparison of allometric change using the size factor and the geometric mean. (DOCX)

S1 Text Data representativeness, comparison of datasets and robustness of statistical analyses. Size, shape and allometry. Comparison between our Factor Analysis and Geometric Morphometrics.
(DOCX)
