# Supplementary material for: Neurocranium versus Face: A Morphometric Approach with Classical Anthropometric Variables for Characterizing Patterns of Cranial Integration in Extant Hominoids and Extinct Hominins
Source: PLoS One. 2015 Jul 15;10(7):e0131055. doi: 10.1371/journal.pone.0131055 (PMC4503590; doi:10.1371/journal.pone.0131055)

**S1 Figure.** **Principal components analysis of craniometric variation in the two samples compared (Howells dataset and this study).** Eigenvectors extracted from the correlation matrix for the six variables analyzed (see Table S3), which were logarithmically transformed prior to analysis. The graph shows the specimens' scores on the two first components.


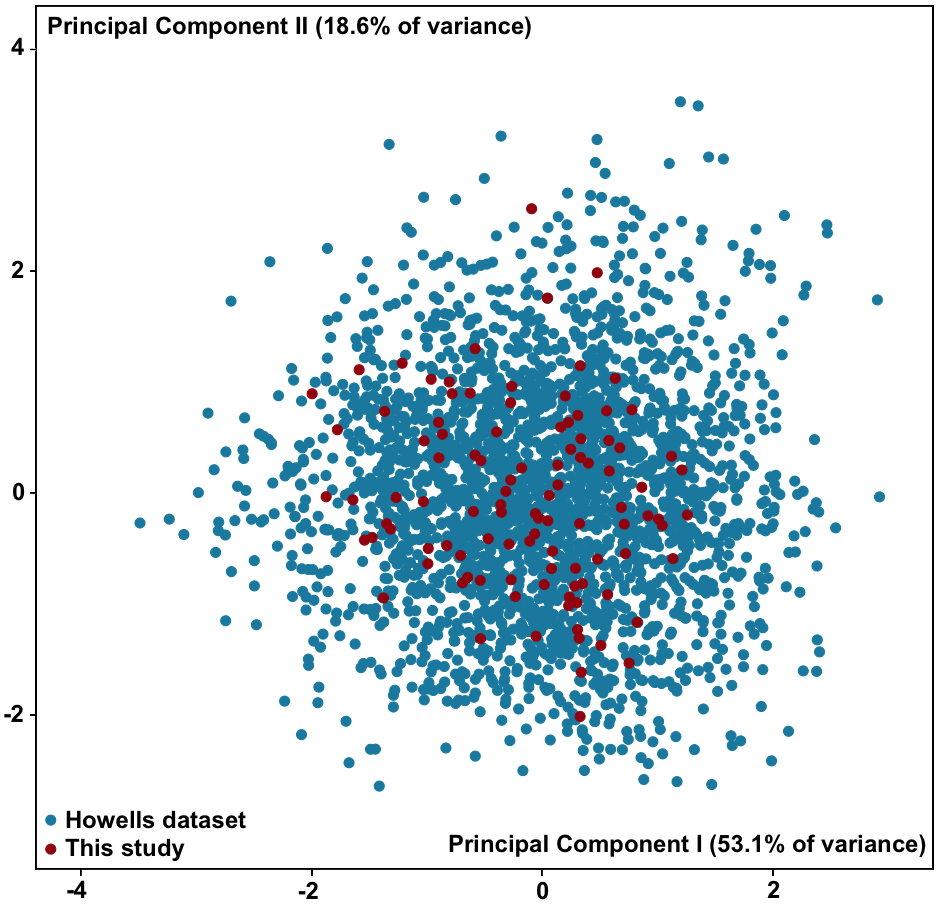

Supplement: S1 Fig — (DOCX) [file pone.0131055.s001.docx]
