# Supplementary material for: Neurocranium versus Face: A Morphometric Approach with Classical Anthropometric Variables for Characterizing Patterns of Cranial Integration in Extant Hominoids and Extinct Hominins
Source: PLoS One. 2015 Jul 15;10(7):e0131055. doi: 10.1371/journal.pone.0131055 (PMC4503590; doi:10.1371/journal.pone.0131055)

**S2 Figure.** Principal components analysis of the hominoid cranial dataset analyzed (black points and colored squares) showing the projections of selected fossils for which alternative measurements of the variables are available (yellow circles: OH5; green circles: SK48; light blue circles: Sts5; dark blue circles: ER1813; pink: Stw53; data from Table S4).


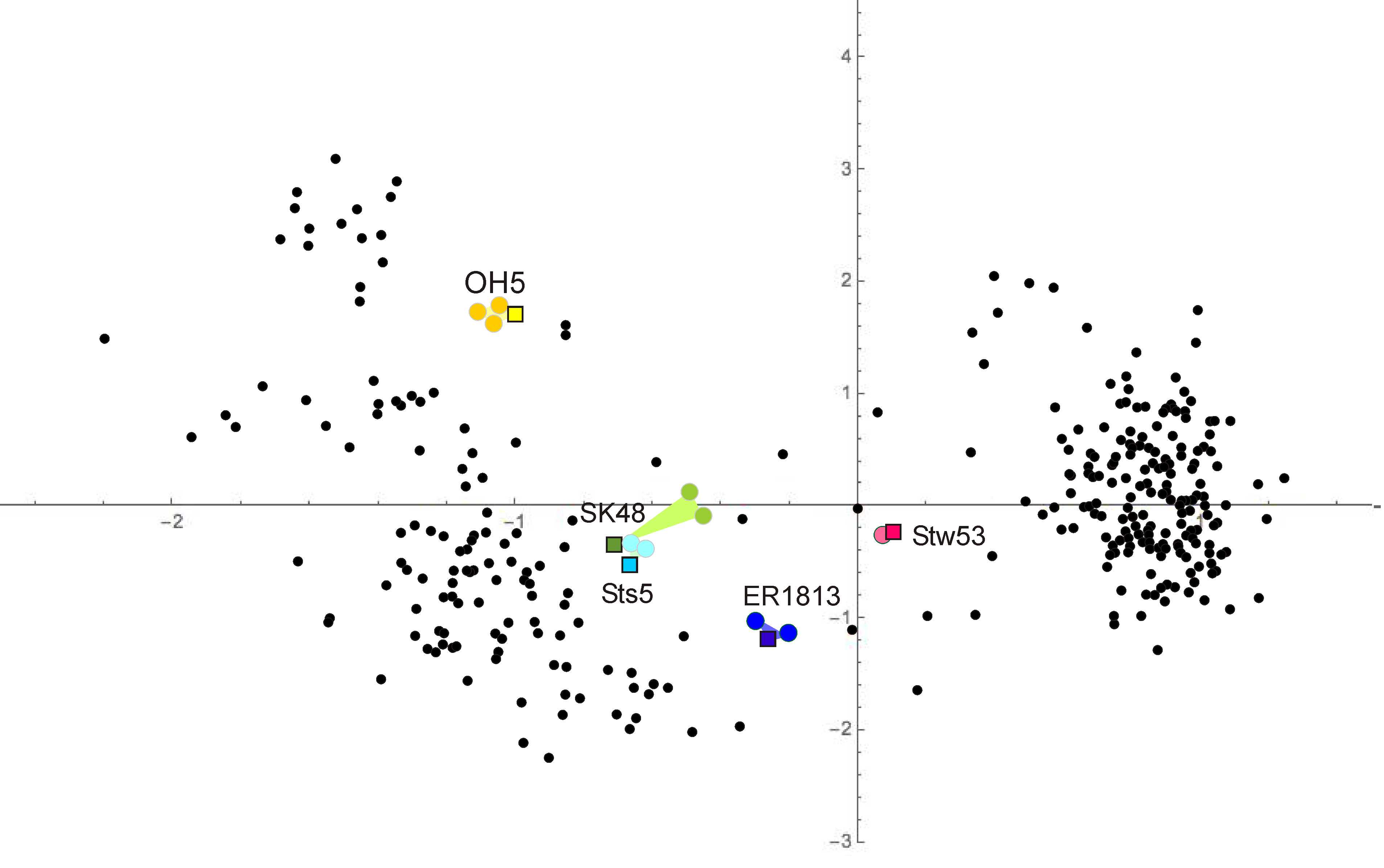

Supplement: S2 Fig — (DOCX) [file pone.0131055.s002.docx]
