# Supplementary material for: Neurocranium versus Face: A Morphometric Approach with Classical Anthropometric Variables for Characterizing Patterns of Cranial Integration in Extant Hominoids and Extinct Hominins
Source: PLoS One. 2015 Jul 15;10(7):e0131055. doi: 10.1371/journal.pone.0131055 (PMC4503590; doi:10.1371/journal.pone.0131055)

**S3 Figure.** Principal components analysis of the hominoid cranial dataset including for each fossil hominin crania 500 simulations in which the original measurements were randomly varied up to 5% according to a uniform distribution (green points). Blue circles: projections for the living species. Red circles: projections for the fossil hominins in the original analysis.


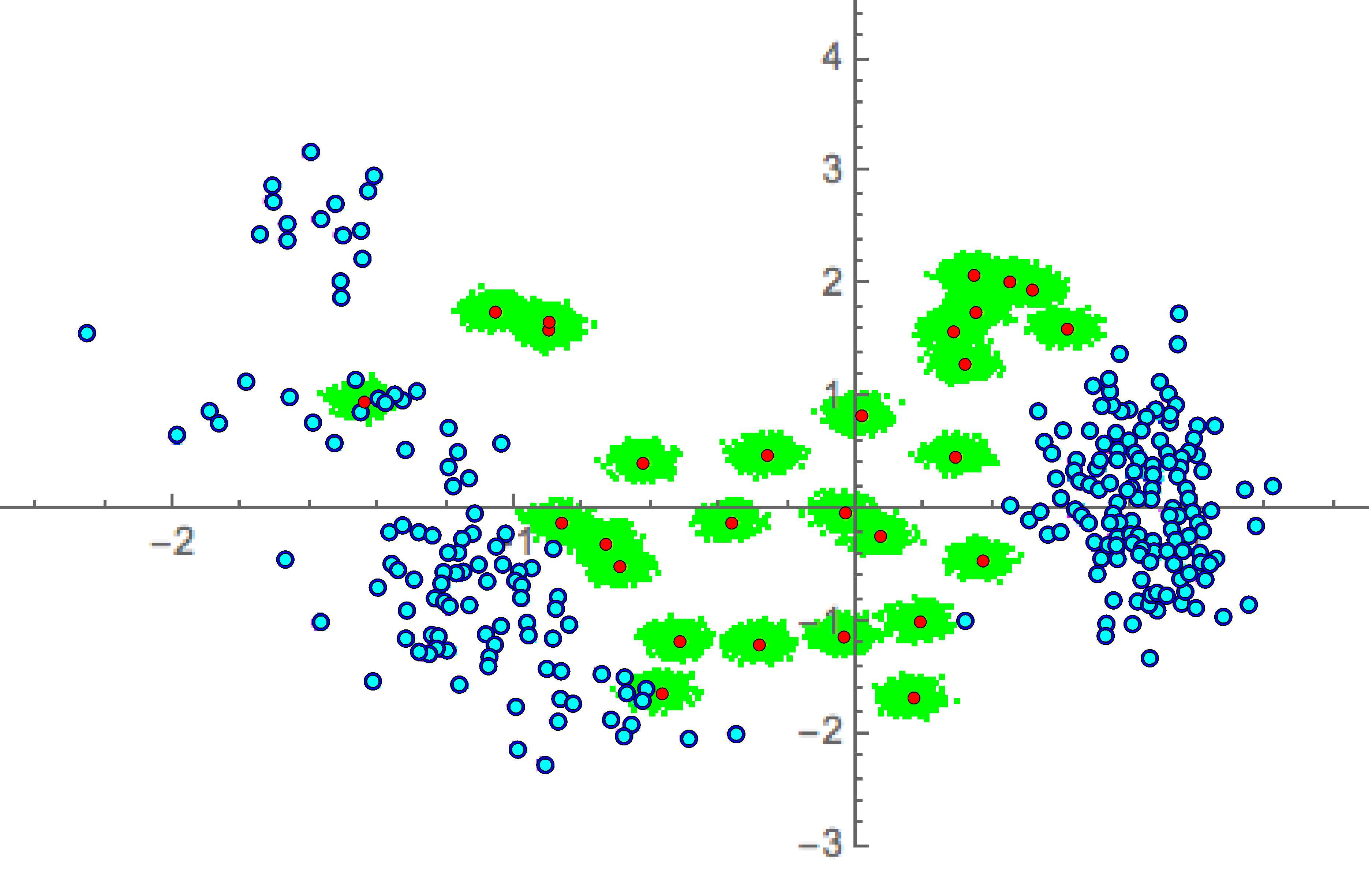

Supplement: S3 Fig — (DOCX) [file pone.0131055.s003.docx]
