# Supplementary material for: Neurocranium versus Face: A Morphometric Approach with Classical Anthropometric Variables for Characterizing Patterns of Cranial Integration in Extant Hominoids and Extinct Hominins
Source: PLoS One. 2015 Jul 15;10(7):e0131055. doi: 10.1371/journal.pone.0131055 (PMC4503590; doi:10.1371/journal.pone.0131055)

**S4 Figure.** **Comparison between the principal components of Guy et al. and the factor analysis of this study.** A) Plot of scores for FI vs. PCI. B) Plot for centroid size of Guy et al. on FII scores.


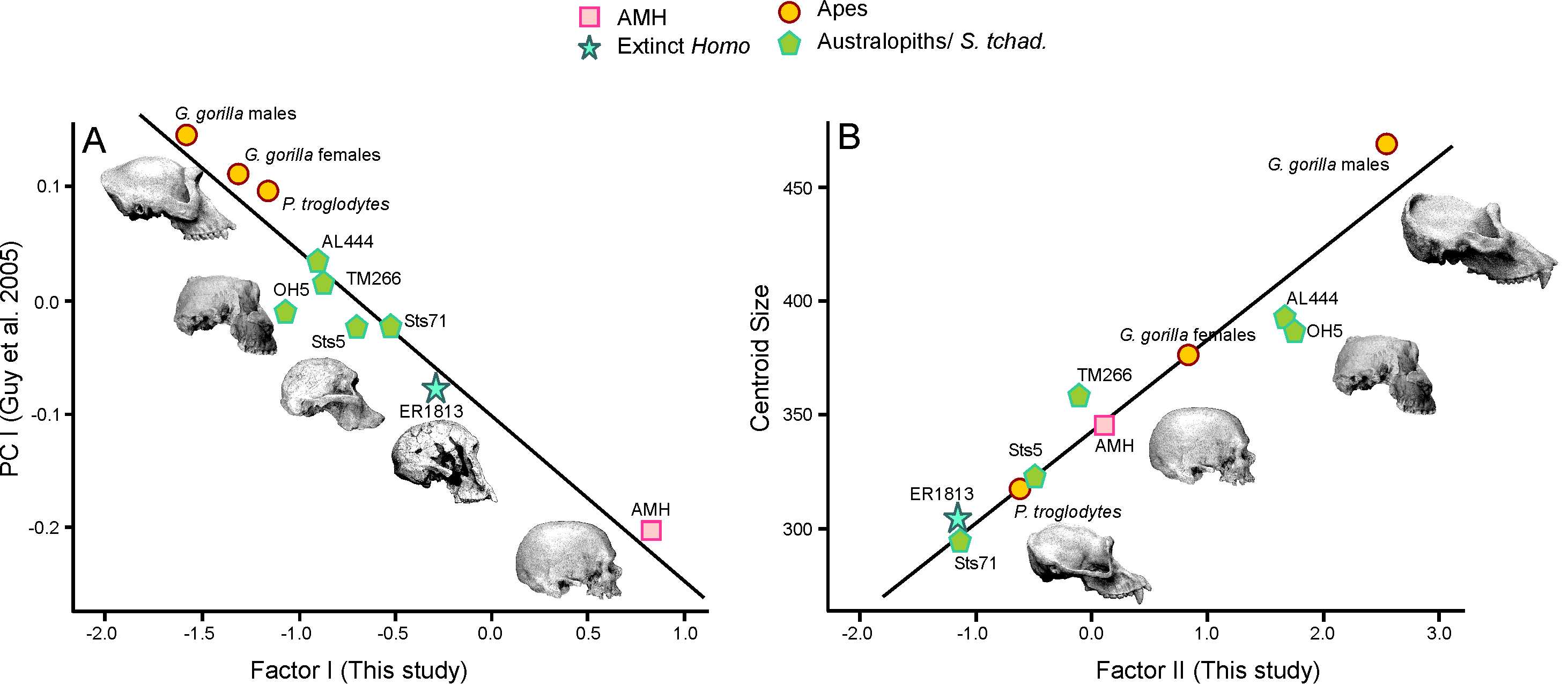

Supplement: S4 Fig — (DOCX) [file pone.0131055.s004.docx]
