# Supplementary material for: Neurocranium versus Face: A Morphometric Approach with Classical Anthropometric Variables for Characterizing Patterns of Cranial Integration in Extant Hominoids and Extinct Hominins
Source: PLoS One. 2015 Jul 15;10(7):e0131055. doi: 10.1371/journal.pone.0131055 (PMC4503590; doi:10.1371/journal.pone.0131055)
Supplement: S1 Table — (DOCX) [file pone.0131055.s005.docx]

**S1 Table. Measurements of fossil hominin crania.** References for source data in brackets. Variables: glabella-opistocranion length (GOL), basion-bregma height (BBH), maximum biparietal cranial breadth (XCB), basion-prosthion length (BPL), nasion-prosthion height (NPH) and bizygomatic breadth (ZYB). (*) ZYB estimated on a cast as the two times the distance from the most external point of the zygomatic arch in frontal view to the middle line of the cranium. (**) Measurement taken on a cast. (+) Measurement taken on a virtual reconstruction [21]. Those measurements taken from photographs appear in bold type.

| **Individual** | **Abbreviation** | **GOL** | **BBH** | **XCB** | **NPH** | **BPL** | **ZYB** |
| --- | --- | --- | --- | --- | --- | --- | --- |
| **TM 266-01-060-1** | TM266 | 173.0 [1] | 86.0 [1] | **92.8** | 75.0 [1] | **132.9** | **132.8** |
| **AL 444-2** | AL444-2 | 167.0 [2] | 102.0 [2] | 122.0 [2] | 100.0 [2] | **145.0** | 167.0 [2] |
| **Sts 5** | Sts5 | 146.0 [3] | 101.0 [3] | 98.0 [3] | 77.0 [3] | 127.0 [3] | 126.0 [3] |
| **Sts 71** | Sts71 | 129.0 [3] | 88.0 (+) | 108.0 [3] | 71.0 [3] | 106.0 (+) | 126.0 [3] |
| **KNM-WT 17000** | WT17000 | 148.5 (**) | 93.1 (**) | 100.0 [3] | 99.0 [3] | 149.4 (**) | 162 (**) |
| **KNM-ER 406** | ER406 | 163.0 [3] | 103.0 [3] | 112.0 [3] | 88.0 [3] | 135.0 [3] | 179.0 [3] |
| **OH 5** | OH5 | 168.5 [4] | 100.7 [4] | 108.8 [4] | 108.0 [4] | 134.6 [4] | 169.4 [4] |
| **SK 48** | SK48 | 140.4 (**) | 91.0 (**) | 100.0 [3] | 80.0 [3] | 97.0 (**) | 145.0 (**) |
| **DNH 7** | DNH7 | **140.5** | **88.8** | **89.3** | **71.2** | **98.2** | **112.7** |
| **KNM-ER 1813** | ER1813 | 145.0 [20] | 94.8 [20] | 102.8 [20] | 66.0 [20] | 98.3 [20] | 116.8 [20] |
| **OH 24** | OH24 | 145.0 [3] | 89.0 [3] | 118.0 [3] | 67.0 [3] | 92.0 [3] | **120.0** |
| **KNM-ER 1470** | ER1470 | 166.0 [3] | 104.0 [5] | 126.0 [3] | 90.0 [3] | 115.0 [6] | 135.0 [6] |
| **D 2282** | D2282 | 163.0 [7] | 92.0 [6] | 116.0 [7] | 81.0 [6] | 110.0 [6] | 130.0 [8] |
| **D 2700** | D2700 | 155.0 [7] | 100.0 [6] | 117.0 [7] | 63.0 [9] | 100.0 [6] | 116.0 [6] |
| **D 3444** | D3444 | 163.0 [8] | **107.0** | 122.0 [8] | **63.0** | **93.8** | **126.7** |
| **D 4500** | D4500 | 165.0 [19] | 92.0 [19] | 109.0 [19] | 73.0 [19] | 127.0 [19] | 149.0 [19] |
| **KNM-ER 3733** | ER3733 | 182.0 [3] | 108.0 [3] | 135.0 [3] | 83.0 [3] | 120.0 [3] | 138.0 [3] |
| **KNM-WT 15000** | WT15000 | 175.0 [6] | 101.0 [6] | 124.0 [6] | 78.0 [6] | 118.0 [6] | 121.6 [6] |
| **Stw 53** | Stw53 | 167.0 [10] | 110.0 [10] | 108.0 [10] | 70.0 [10] | **97.0** | **130.0** |
| **Sangiran 17** | Sang17 | 206.0 [3] | 110.0 [3] | 150.0 [3] | 82.0 [3] | 118.0 [3] | 150.0 [3] |
| **Kabwe** | Kabwe | 210.0 [11] | 129.0 [11] | 144.0 [11] | 95.0 [11] | 115.0 [11] | 144.5 (*) |
| **SH Cranium 5** | SH5 | 185.0 [12] | 125.0 [12] | 146.5 [12] | 85.0 [12] | 115.3 [12] | 144.0 [12] |
| **Steinheim** | Steinh | 185.0 [13] | 110.0 [13] | 132.5 [13] | 74.5 [13] | 109.0 [13] | 132.0 [13] |
| **Petralona** | Petr | 208.0 [14] | 127.0 [14] | 152.0 [14] | 90.0 [14] | 119.0 [14] | 157.0 [14] |
| **Shanidar I** | Shan1 | 207.0 [15] | 135.0 [15] | 154.0 [15] | 86.0 [15] | 117.1 [15] | 140.0 [15] |
| **La Chapelle** | LaCh | 208.0 [11] | 131.0 [11] | 156.0 [11] | 86.0 [11] | 124.8 [11] | 153.0 [11] |
| **La Ferrasie I** | LaFerr1 | 207.5 [16] | 135.0 [16] | 158.0 [17] | 88.0 (m) | 124.0 [16] | 148.5 [16] |
| **LB-1** | LB1 | 143.0 [18] | 89.0 [18] | 110.0 [18] | 53.0 [18] | 88.0 [18] | 117.0 (**) |

**References**

1. Zollikofer CPE, Ponce de Leon MS, Lieberman DE, Guy F, Pilbeam D, Likius A, Mackaye HT, Vignaud P, Brunet M. Virtual cranial reconstruction of *Sahelanthropus tchadensis*. Nature. 2005;434: 755-759.

2. Kimbel WH, Johanson DC, Rak Y. The skull of *Australopithecus afarensis*. Oxford: Oxford University Press; 2004.

3. Wood BA. Hominid cranial remains. Koobi For. Research Project 4. Oxford: Clarendon; 1991.

4. Benazzi S, Bookstein FL, Strait DS, Weber GW. A new OH5 reconstruction with an assessment of its uncertainty. J Hum Evol. 2011;61: 75-88.

5. Hawks J, Wolpoff MH. Endocranial Capacity of Early Hominids. Science.1999;283: 5398.

6. de Lumley MA, Gabounia L, Vekua A, Lordkipanidze D. Les restes humains du Pliocene final et du debut du Pleistocene inferieur de Dmanissi, Georgie (1991-2000). I-Les cranes, D 2280, D 2282, D 2700. L'Anthropologie. 2006;110: 1-110.

7. Lordkipanidze D, Vekua A, Ferring R, Rightmire GP, Agusti J, Kiladze G, Mouskhelishvili A, Nioradze M, de Leon MSP, Tappen M, Zollikofer CPE. The earliest toothless hominin skull. Nature. 2005; 434: 717-718.

8. Gabunia L, Vekua A, Lordkipanidze D, Swisher CC, III, Ferring R, Justus A, Nioradze M, Tvalchrelidze M, Anton SC, Bosinski G, Joris O, Lumley M-Ad, Majsuradze G, Mouskhelishvili A. Earliest Pleistocene Hominid Cranial Remains from Dmanisi, Republic of Georgia: Taxonomy, Geological Setting, and Age. Science. 2000; 288:1019-1025.

9. Vekua A, Lordkipanidze D, Rightmire GP, Agusti J, Ferring R, Maisuradze G, Mouskhelishvili A, Nioradze M, de Leon MP, Tappen M, Tvalchrelidze M, Zollikofer C. A New Skull of Early *Homo* from Dmanisi, Georgia. Science. 2002;297: 85-89.

10. Curnoe D, Tobias PV. Description, new reconstruction, comparative anatomy, and classification of the Sterkfontein Stw 53 cranium, with discussions about the taxonomy of other southern African early *Homo* remains. J Hum Evol. 2006;50: 36-77.

11. Weidenreich F. The skull of *Sinanthropus pekinensis*: a comparative study on a primitive hominid skull. Palaeontol. Sin. 1943;D10: 1−485.

12. Arsuaga JL, Martinez I, Gracia A, Lorenzo C. The Sima de los Huesos crania (Sierra de Atapuerca, Spain). A comparative study. J Hum Evol. 1997;33: 219-281.

13. Howell FC. European and Northwest African Middle Pleistocene Hominids. Curr Anthropol. 1960;1: 195-232.

14. Murrill RI. Petralona Man. A Descriptive and Comparative Study, with New Information on Rhodesian Man. Springfield: Charles C. Thomas Pub. Ltd; 1981.

15. Trinkaus E. The Shanidar Neandertals. Academic Press, New York; 1983.

16. Vandermeersch B. Les hommes fossiles de Qafzeh (Israel). Paris: Centre National de la Recherche Scientifique; 1981.

17. Suzuki H, Takai F. The Amud Man and His Cave Site. Tokyo: University of Tokyo; 1970.

18. Brown P, Sutikna T, Morwood MJ, Soejono RP, Jatmiko, Wayhu Saptomo E, Awe Due R. A new small-bodied hominin from the Late Pleistocene of Flores, Indonesia. Nature. 2004; 431: 1055-1061.

19. Lordkipanidze D, Ponce de León, MS, Margvelashvili A, Rak Y, Rightmire GP, Vekua A, Zollikofer CPE. A complete skull from Dmanisi, Georgia, and the evolutionary biology of early *Homo*. Science. 2013; 342: 326-331.

20. Benazzi S, Gruppioni G, Strait DS, Hublin JJ. Technical Note: Virtual reconstruction of KNM-ER 1813 *Homo habilis* cranium. Am J Phys Anthropol. 2014; 153: 154-160.

21. Weber GW , Seidler H, Thackeray F, Braga J, Treil J, Recheis W, zur Nedden D, Conroy GC. Sts 71. CD-ROM Edition - Fossil Hominids, Vienna: Inst. for Anthropology, University of Vienna. 2002.
