# Supplementary material for: Neurocranium versus Face: A Morphometric Approach with Classical Anthropometric Variables for Characterizing Patterns of Cranial Integration in Extant Hominoids and Extinct Hominins
Source: PLoS One. 2015 Jul 15;10(7):e0131055. doi: 10.1371/journal.pone.0131055 (PMC4503590; doi:10.1371/journal.pone.0131055)
Supplement: S2 Table — (DOCX) [file pone.0131055.s006.docx]

**S2 Table. Comparison with Howells dataset.** Values of the coefficient *K* of Lubischew and percentages of overlap for the comparison between the means and standard deviations of the six craniometric measurements used for anatomically modern humans in this study and their values in Howells dataset. For abbreviations of variables, see Table S1.

| **Craneometric variables** | **Howell's database (N=2254)** | | **This study**  **(N=103)** | | **√K/2** | **Overlap** |
| --- | --- | --- | --- | --- | --- | --- |
|  | **Mean± Sd** | **Min-Max** | **Mean± Sd** | **Min-Max** |  |  |
| **GOL** | 179.17 ±8.54 | 151-206 | 179.28±7.01 | 164-194 | 0.000084 | 99.99% |
| **BBH** | 131.64±7.24 | 107-155 | 132.97±6.30 | 119-148 | 0.017054 | 98.64% |
| **XCB** | 136.85±7.29 | 116-167 | 134.67±5.63 | 126-152 | 0.045515 | 96.37% |
| **ZYB** | 130.77±7.79 | 105-158 | 125.51±6.61 | 112-142 | 0.230788 | 81.75% |
| **BPL** | 97.78±6.38 | 80-123 | 95.70±5.55 | 83-109 | 0.053715 | 95.72% |
| **NPH** | 65.98±5.54 | 48-82 | 68.24±5.92 | 58-86 | 0.082696 | 93.41% |
