# Supplementary material for: Neurocranium versus Face: A Morphometric Approach with Classical Anthropometric Variables for Characterizing Patterns of Cranial Integration in Extant Hominoids and Extinct Hominins
Source: PLoS One. 2015 Jul 15;10(7):e0131055. doi: 10.1371/journal.pone.0131055 (PMC4503590; doi:10.1371/journal.pone.0131055)
Supplement: S3 Table — (DOCX) [file pone.0131055.s007.docx]

**S3 Table. Principal components analysis of craniometric variables in our sample of *Homo sapiens* and Howells dataset.** Eigenvalues (λ), percentages of variance explained by the first two principal components (% var.) and factor loadings of the craniometric variables on them.

| Variables | PCI | PCII |
| --- | --- | --- |
| Log_10_GOL | 0.775 | -0.329 |
| Log_10_BBH | 0.752 | -0.085 |
| Log_10_XCB | 0.566 | 0.717 |
| Log_10_ZYB | 0.855 | 0.170 |
| Log_10_BPL | 0.637 | -0.635 |
| Log_10_NPH | 0.751 | 0.229 |
| λ | 3,188 | 53,126 |
| % var. | 1,114 | 18,565 |
