# Supplementary material for: Neurocranium versus Face: A Morphometric Approach with Classical Anthropometric Variables for Characterizing Patterns of Cranial Integration in Extant Hominoids and Extinct Hominins
Source: PLoS One. 2015 Jul 15;10(7):e0131055. doi: 10.1371/journal.pone.0131055 (PMC4503590; doi:10.1371/journal.pone.0131055)
Supplement: S4 Table — (DOCX) [file pone.0131055.s008.docx]

**S4 Table.** Measurements for several hominin crania from different sources.

| **Specimen** | **GOL** | **XCB** | **BBH** | **BPL** | **NPH** | **ZYG** | **Ref.** |
| --- | --- | --- | --- | --- | --- | --- | --- |
| OH5 | 173.0 | 110.0 | 98.0 | 137.0 | 111.5 | 168.0 | 1 |
| OH5 | 173.0 | 116.0 | 98.0 | 137.0 | 112.0 | 168.0 | 2 |
| OH5 | 168.5 | 108.8 | 100.7 | 134.6 | 108.0 | 169.4 | 3 |
| SK48 | -- | 110.0 | -- | -- | 80.0 | -- | 2 |
| SK48 | 170.0 | -- | -- | -- | 80.0 | 146.0 | 1 |
| Sts5 | 146.0 | 98.0 | 101.0 | 127.0 | 77.0 | 127.0 | 2 |
| Sts5 | 146.8 | 96.5 | 100.0 | 122.0 | 75.0 | 127.0 | 1 |
| ER 1813 | 149.0 | 104.0 | 90.0 | 105.0 | 66.0 | 117.0 | 2 |
| ER 1813 | 145.0 | 102.8 | 94.8 | 98.3 | 66.0 | 116.8 | 3 |
| Stw53 | 167.0 | 106.0 | 110.0 | 70.0 | -- | -- | 4 |

**References**

1. Tobias PV.. Olduvai Gorge. The craniium of *Australopithecus* (*Zinjanthropus*) *boisei:* Cambridge University Press, Cambrigde. 1967.

2. Wood BA. Koobi Fora Research Project, Vol. 4. Hominid Cranial Remains: Oxford University Press, New York. 1991

3. Benazzi S, Bookstein FL, Strait D S, Weber GW. A new OH5 reconstruction with an assessment of its uncertainty. J. Hum. Evol. 2011; 61: 75–88.

4. Curnoe D, Tobias PV. Description, new reconstruction, comparative anatomy, and classification of the Sterkfontein Stw 53 cranium, with discussions about the taxonomy of other southern African early *Homo* remains. J. Hum. Evol. 2006; 50: 36–77.
