# Supplementary material for: Neurocranium versus Face: A Morphometric Approach with Classical Anthropometric Variables for Characterizing Patterns of Cranial Integration in Extant Hominoids and Extinct Hominins
Source: PLoS One. 2015 Jul 15;10(7):e0131055. doi: 10.1371/journal.pone.0131055 (PMC4503590; doi:10.1371/journal.pone.0131055)
Supplement: S5 Table — (DOCX) [file pone.0131055.s009.docx]

**S5 Table:** Reduced major axis regressions for the scores on the first factor (shape) on the logarithm of the geometric mean of the six original measurements in different hominoid groups. *R^2^:* coefficient of determination; *p*: probability for *r =* 0; n.s.: non significant (i.e., *p >* 0.05). Bstr95%: bootstrapped 95% confidence intervals for the slopes (2,000 replicates). Australopiths* refers to all australopith crania except WT17000. AMH: anatomically modern humans.

| **Group** | ***N*** | ***R^2^*** | **Slope** | **Bstr95%** | ***p* ( *r =* 0)** |
| --- | --- | --- | --- | --- | --- |
| AMH | 174 | 0.039 | -6.225 | [-7.099; -5.284] | 0.0089 |
| *Pan paniscus* | 20 | 0.033 | -15.058 | [-48.39; -9.394] | n.s. |
| *Pan troglodytes* | 54 | 0.013 | -10.124 | [-31.69; -7.344] | n.s. |
| *Gorilla gorilla* | 29 | 0.578 | -6.890 | [-8.597; -5.17] | 0.0002 |
| *G. gorilla* ♂ | 15 | 0.034 | -8.842 | [-31.35; -4.475] | n.s. |
| *G. gorilla* ♀ | 14 | 0.360 | -13.777 | [-19.5; -5.417] | 0.0233 |
| *Pongo pygmaeus* | 14 | 0.732 | -7.403 | [-9.462; -5.935] | <0.00001 |
| *P. pygmaeus* ♂ | 7 | 0.499 | -18.639 | [-51.37; 5.864] | n.s. |
| *P. pygmaeus* ♀ | 7 | 0.817 | -13.181 | [-17.67; -6.103] | 0.0052 |
| Great apes | 117 | 0.522 | -6.109 | [-7.059; -5.007] | <0.00001 |
| African apes | 103 | 0.679 | 5.092 | [-5.647; -4.495] | <0.00001 |
| Australopithecines | 9 | 0.500 | -5.800 | [-8.864; -1.517] | 0.0332 |
| Australopithecines* | 8 | 0.831 | -7.315 | [-9.216; -4.324] | 0.0016 |
| Extinct *Homo* | 19 | 0.286 | 6.4659 | [3.636; 8.989] | 0.0184 |
