# Supplementary material for: Neurocranium versus Face: A Morphometric Approach with Classical Anthropometric Variables for Characterizing Patterns of Cranial Integration in Extant Hominoids and Extinct Hominins
Source: PLoS One. 2015 Jul 15;10(7):e0131055. doi: 10.1371/journal.pone.0131055 (PMC4503590; doi:10.1371/journal.pone.0131055)
Supplement: S6 Table — (DOCX) [file pone.0131055.s010.docx]

**S6 Table:** Reduced major axis regressions of the scores on the first factor (shape) on the second factor (left) and on the standardized logarithms of the geometric mean of the six original measurements (right) in different hominoid groups. *R^2^:* coefficient of determination; *p*: probability for *r =* 0; n.s.: non significant (i.e., *p >* 0.05). Bstr95%: bootstrapped 95% confidence intervals for the slopes (2,000 replicates). Australopiths* refers to all australopith crania except WT17000. AMH: anatomically modern humans.

|  |  |  |  | **Factor I on Factor II** |  |  | **Factor I on Estandardized LogGM** | |  |
| --- | --- | --- | --- | --- | --- | --- | --- | --- | --- |
| **Group** | **N** | **R^2^** | **Slope** | **Bstr95%** | ***p* ( r = 0)** | **R^2^** | **Slope** | **Bstr95%** | ***p* ( r = 0)** |
| **AMH** | 174 | 0.024 | -0.247 | [-0.286; -0.208] | 0.0418 | 0.039 | -0.236 | [-0.269; -0.200] | 0.0089 |
| ***Pan paniscus*** | 20 | 0.040 | -0.493 | [-1.583; -0.255] | n.s. | 0.033 | -0.570 | [-1.818; -0.366] | n.s. |
| ***Pan troglodytes*** | 54 | 0.011 | -0.370 | [-1.161; -0.267] | n.s. | 0.013 | -0.383 | [-1.209; -0.283] | n.s. |
| ***Gorilla gorilla*** | 29 | 0.553 | -0.197 | [-0.237; -0.155] | <0.00001 | 0.554 | -0.211 | [-0.257; -0.166] | <0.00001 |
| ***G. gorilla* ♂** | 15 | 0.016 | -0.327 | [-1.174; -0.167] | n.s. | 0.034 | -0.335 | [-1.188; -0.186] | n.s. |
| ***G. gorilla* ♀** | 14 | 0.487 | -0.574 | [-0.809; -0.273] | 0.0055 | 0.360 | -0.521 | [-0.744; -0.198] | 0.0233 |
| ***Pongo pygmaeus*** | 14 | 0.709 | -0.274 | [-0.360; -0.217] | 0.0002 | 0.732 | -0.280 | [-0.356; -0.225] | 0.0001 |
| ***P. pygmaeus* ♂** | 7 | 0.490 | -0.725 | [-2.509; 0.425] | n.s. | 0.499 | -0.705 | [-1.973; 0.319] | n.s. |
| ***P. pygmaeus* ♀** | 7 | 0.664 | -0.505 | [-0.720; -0.145] | 0.0255 | 0.817 | -0.499 | [-0.664; -0.240] | 0.0052 |
| **Great apes** | 117 | 0.512 | -0.233 | [-0.269; -0.1912] | <0.00001 | 0.522 | -0.231 | [-0.266; -0.189] | <0.00001 |
| **African apes** | 103 | 0.668 | -0.195 | [-0.218; -0.168] | <0.00001 | 0.679 | -0.193 | [-0.214; -0.169] | <0.00001 |
| **Australopithecines** | 9 | 0.500 | -0.220 | [-0.337; -0.055] | 0.0331 | 0.500 | -0.219 | [-0.334; -0.062] | 0.0332 |
| **Australopithecines*** | 8 | 0.831 | -0.137 | [-0.178; -0.082] | 0.0016 | 0.810 | -0.137 | [-0.180; -0.084] | 0.0023 |
| **Extinct Homo** | 19 | 0.259 | 0.277 | [0.148; 0.386] | 0.0260 | 0.286 | 0.245 | [0.138; 0.340] | 0.0184 |
