# Supplementary material for: Neurocranium versus Face: A Morphometric Approach with Classical Anthropometric Variables for Characterizing Patterns of Cranial Integration in Extant Hominoids and Extinct Hominins
Source: PLoS One. 2015 Jul 15;10(7):e0131055. doi: 10.1371/journal.pone.0131055 (PMC4503590; doi:10.1371/journal.pone.0131055)
Supplement: S1 Text — Size, shape and allometry. Comparison between our Factor Analysis and Geometric Morphometrics. (DOCX) [file pone.0131055.s011.docx]

**S1 Text.**

**Data representativeness, comparison of datasets and robustness of statistical analyses**

S1 Table includes the measurements taken on the fossil crania of hominins.

Data representativeness was tested by a comparison of our sample of anatomically modern humans (AMH, *H. sapiens*) used in this study with data included in Howells craniometric dataset (http://web.utk.edu/~auerbach/HOWL.htm), which comprises measurements taken in 2,524 human crania from 28 populations. Unfortunately, there is no equivalent dataset available for the great apes.

The comparison of our sample of *H. sapiens* with Howells dataset was performed calculating in both datasets the mean values and standard deviations of the six craniometric variables. Then, the degree of overlap between both distributions for each variable was estimated quantitatively with the method of Lubischew [1], which allows testing the power of single characters as discriminators [2-4]. The coefficient of discrimination is K = (X_A_ – X_B_)^2^/2S_D_^2^, where *X_A_* is the mean of character *X* for sample *A, X_B_* is the corresponding mean for sample *B,* and *S_x_^2^* is the pooled variance of character *X* in both samples [S_D_^2^ = (σx_A_^2^n_A_ + x_B_^2^n_B_)/(n_A_ + n_B_), where *σx_A_^2^* and *σx_B_^2^* are the variances of character *X* for samples *A* and *B,* and *n_A_* and *n_B_* are their sample sizes, respectively]. Given that the percentage of misclassifications with variable *X* is approximately the probability that a normal deviate exceeds *√K/2,* the greater *K* is the better character *X* is as a discriminator. For example, 95% of identifications will be correct with *K =* 7.68, which means a degree of overlap between the populations compared of only 5% (two-tailed *t-*test, which requires the distribution of variable *X* in both samples to be approximately normal and of equal variance, as in this case).

S2 Table shows the values of *√K/2* and their translation into percentages of overlap between the distributions for each variable, which are greater than 80% in all cases and are in excess of 90% for five out of the six variables. This indicates that, compared with Howells dataset, our sample of human crania is not biased.

In addition, a principal components analysis was performed over the values of the log-transformed craniometric variables joining our sample of *H. sapiens* with Howells dataset, in order to evaluate the patterns of morphospace occupation by both samples. S1 Figure shows the scores of human crania on the first two components, which jointly account for >70% of the original variance. According to the factor scores of the variables on these axes (S3 Table), PCI can be interpreted in an *ad-hoc* manner as a size vector (all variables load positively) while PCII is a shape vector (XCB, ZYG and NPH load positively while GOL, BBH and BPL load negatively). The specimens of both datasets score similarly on this plot, although the sample from Howells shows greater morphological variability on the two axes (the ranges of PCI scores for Howells dataset and our study are -3.49 to 2.93 and -2.00 to 1.26, respectively; the corresponding figures for PCII are: -2.64 to 3.53 and -2.01 to 2.56, respectively). However, this merely reflects the larger size of Howells sample, which incorporates crania from a high number of human populations.

In the case of the extinct hominins, our sample comprises those cranial specimens whose preservational completeness allowed measuring the six variables used in this study. In addition, whenever it was possible the measurements taken were those that appeared in the original descriptions and/or in additional reconstructions. This precluded any comparison with other datasets. However, we used two approaches for testing the robustness of our analyses on these cranial specimens. First, in those cases in which other craniometric variables were available (S4 Table), these were employed as independent case studies for evaluating the consistency between their scores on the principal components and our own data; when only some measurements were available for a given specimen, the estimates used for the other variables were those of the original analysis. Results obtained (S2 Fig.) showed that the projections for the same specimen were always in close proximity.

The second approach consisted of the use of 500 generated simulations for each fossil cranium in which all the original measurements were varied simultaneously at random up to 5% according to a uniform distribution. The projection of their scores on the principal components plot (S3 Fig.) shows that these simulations clustered around the projections of our original data in all cases, which argues again in favor of the robustness of the analysis.

**Size, shape and allometry**

The concept of allometry has developed over time and there are currently two major conceptual frameworks of allometry [5]: the Huxley-Jolicoeur school, which proposed the use of the first principal component of the log-transformed variables as a size vector, and the Gould-Mosimann school, which chose the geometric mean of all variables as the size variable. In this article, we have used the first approach in the search for allometries. However, we have also tested for shaped differences using the second approach (S5 Table). S6 Table shows the comparison between these methods after standardization of variables. The results obtained are quite similar in both cases.

**Comparison between our Factor Analysis and Geometric Morphometrics**

In an attempt to evaluate to which extent the first axis captures the general aspects of cranial shape derived from the six osteological variables, the scores of the specimens on this factor were plotted against the scores on the first principal component obtained by Guy et al. [6] using geometric morphometric methods with three-dimensional landmarks. There are six fossil specimens whose scores can be directly compared in the two analyses. In addition, the mean scores on both analyses for AMH, *P. troglodytes* and *G. gorilla* males and females were used, in order to cover the whole morphological spectrum of hominoid crania analyzed. The plot obtained (S4 Fig. A) shows an approximately linear relationship between both sets of scores, with a correlation of *r =* -0.959 (*p <* 1.2 10^-5^), which suggests that there is a reasonable correspondence between both axes. The correlation between the centroid sizes and the scores on the second factor (i.e., the size vector) for the observations in common with [6] is *r =* 0.958 (*p* < 1.3 10^-5^) (S4 Fig. B).

**References**

1. Lubischew AA. On the use of discriminant functions in taxonomy. Biometrics. 1962; 18: 455–477.

2. Jiménez-Arenas JM, Palmqvist P, Pérez-Claros JA.. A probabilistic approach to the craniometric variability of the genus Homo and inferences on the taxonomic affinities of the first human population dispersing out of Africa. Quatern Int. 2011; 243: 219–230

3. Palmqvist P, Torregrosa V, Pérez-Claros JA, Martínez-Navarro B, Turner A. A re-evaluation of the diversity of *Megantereon* (Mammalia, Carnivora, Machairodontinae) and the problem of species identification in extinct carnivores. J Vert Paleontol. 2007; 27: 160–175.

4. Palmqvist P, González-Donoso JM, De Renzi M. Rectilinear evolution in arvicoline rodents and numerical dating of Iberian Early Pleistocene sites. Quat Sci Rev. 2014; 98: 100–109.

5. Klingenberg CP. Heterochrony and allometry: the analysis of evolutionary change in ontogeny. Biol Rev. 1998; 73: 79–123.

6. Guy F, Lieberman DE, Pilbeam D, Ponce de Leon M, Likius A, Mackaye HT, Vignaud P, Zollikofer C, Brunet M. Morphological affinities of the Sahelanthropus tchadensis (Late Miocene hominid from Chad) cranium. P Natl Acad Sci USA. 2005; 102: 18836–18841.
